# Negative pressure wound therapy versus usual care for surgical wounds healing by secondary intention (SWHSI-2 trial): study protocol for a pragmatic, multicentre, cross surgical specialty, randomised controlled trial

**DOI:** 10.1186/s13063-021-05662-2

**Published:** 2021-10-25

**Authors:** Ian Chetter, Catherine Arundel, Belen Corbacho Martin, Catherine Hewitt, Caroline Fairhurst, Kalpita Joshi, Andrew Mott, Sara Rodgers, Pedro Saramago Goncalves, David Torgerson, Jacqueline Wilkinson, Jane Blazeby, Rhiannon Macefield, Stephen Dixon, Eileen Henderson, Angela Oswald, Jo Dumville, Matthew Lee, Thomas Pinkney, Nikki Stubbs, Lyn Wilson

**Affiliations:** York Trials Unit, Department of Health Sciences, Faculty of Science, University of York, Lower Ground Floor ARRC Building, York, YO10 5DD UK

**Keywords:** Surgical wounds, Negative pressure wound therapy, Secondary intention, Wound healing, Randomised controlled trial

## Abstract

**Background:**

The majority of surgical wounds are closed (for example with sutures or staples) and so heal by primary intention. Where closure is not possible, or the wound subsequently breaks down, wounds may be left to heal from the bottom up (healing by secondary intention). Surgical wound healing by secondary intention (SWHSI) frequently presents a significant management challenge. Additional treatments are often required during the course of healing, and thus a significant financial burden is associated with treating these wounds.

Increasingly, negative pressure wound therapy (NPWT) is used in the management of SWHSI. This wound dressing system provides a negative pressure (vacuum) to the wound, removing fluid into a canister, which is believed to be conducive to wound healing. Despite the increasing use of NPWT, there is limited robust evidence for the effectiveness of this device. A well-designed and conducted randomised controlled trial is now required to ascertain if NPWT is a clinically and cost-effective treatment for SWHSI.

**Methods:**

SWHSI-2 is a pragmatic, multi-centre, cross surgical specialty, two arm, parallel group, randomised controlled superiority trial. Adult patients with a SWHSI will be randomised to receive either NPWT or usual care (no NPWT) and will be followed up for 12 months.

The primary outcome will be time to healing (defined as full epithelial cover in absence of a scab) in number of days since randomisation. Secondary outcomes will include key clinical events (hospital admission or discharge, treatment status, reoperation, amputation, antibiotic use and death), wound infection, wound pain, health-related quality of life, health utility and resource use.

**Discussion:**

Given the increasing use of NPWT, despite limited high-quality supporting evidence, the SWHSI-2 Trial will provide robust evidence on the clinical and cost-effectiveness of NPWT in the management of SWHSI.

The SWHSI-2 Trial opened to recruitment in May 2019 and is currently recruiting across 20 participating centres.

**Trial registration:**

ISRCTN 26277546. Prospectively registered on 25 March 2019

**Supplementary Information:**

The online version contains supplementary material available at 10.1186/s13063-021-05662-2.

## Administrative information

Note: the numbers in curly brackets in this protocol refer to SPIRIT checklist item numbers. The order of the items has been modified to group similar items (see http://www.equator-network.org/reporting-guidelines/spirit-2013-statement-defining-standard-protocol-items-for-clinical-trials/).

**Table:** 

Title {1}	Negative pressure wound therapy versus usual care for Surgical Wounds Healing by Secondary Intention (SWHSI-2 Trial): study protocol for a pragmatic, multicentre, cross surgical specialty, randomised controlled trial
Trial registration {2a and 2b}.	ISRCTN Registration Number: 26277546 Prospectively registered on 25.03.2019 Recruitment Infographic SWAT - MRC Hub for Trials Methodology Research SWAT repository #116 Registered 13.04.2020 Retention Thank You Card SWAT - MRC Hub for Trials Methodology Research SWAT repository #119 Registered 13.04.2020
Protocol version {3}	v1.4 31.07.2020
Funding {4}	This project was funded by the National Institute for Health Research (NIHR) Health Technology Assessment Programme (Project Reference: 17/42/94). The views expressed are those of the author(s) and not necessarily those of the NIHR or the Department of Health and Social Care.
Author details {5a}	The SWHSI-2 Trial Investigators^+^ ^+^ See Declarations Section for full list of SWHSI-2 Trial Investigators
Name and contact information for the trial sponsor {5b}	Hull University Teaching Hospitals NHS Trust Office 14, 2^nd^ Floor Daisy Building, Castle Hill Hospital, Hull HU16 5JQ
Role of sponsor {5c}	Study sponsor and funder have had no role in study design nor in the collection, management, analysis or interpretation of data. They will have no role in the writing of associated publications and the decision to submit papers for publication.

## Introduction

### Background and rationale {6a}

More than 10 million surgical operations are performed in the NHS every year [1].Wounds are usually closed by apposing the wound edges — “healing by primary intention”. However, when closure is not possible, or when primarily closed wounds break down (dehisce), they are usually left open to heal from the bottom up, through formation of granulation tissue — “healing by secondary intention”.

Surgical wounds healing by secondary intention (SWHSI) are a common, complex problem. A recent survey estimated the UK prevalence of SWHSI to be 4.1 per 10,000 population and identified colorectal, plastics and vascular as the surgical specialties most commonly associated with SWHSI [2].

Treatment of these wounds presents a significant financial burden to the NHS, with costs of SWHSI estimated to be £1060 per patient, before inclusion of treatment costs [3]. The healing pathway of SWHSI patients is often prolonged and complex with a variety of treatment options, ranging from basic, relatively inexpensive wound dressings (around £441 per month) to complex, expensive treatments such as Negative pressure wound therapy (NPWT, around £1323 per month [4]).

These wounds pose a unique management challenge and difficulties for patients; they can remain open for many months, may require a multitude of treatments, are highly susceptible to infection and may require prolonged hospitalisation and/or further operations [5]. The healing pathway of patients with an open wound is often prolonged, which has a substantial impact on health-related quality of life [6, 7]. In a recent cohort study of 396 people undergoing SWHSI, the median time to healing was 86 days (95% confidence interval (CI) 75 to 130). In specific wound sites (e.g. foot/leg), the duration was almost double that of wounds elsewhere on the body [8]. Infection (32.1%), hospital readmission (24.7%) and further surgical procedures (16.8%) were all common [8]. People with SWHSI often require frequent dressing changes, and for many years standard dressings that filled cavities and/or covered open surgical wounds were used widely. More recently negative pressure wound therapy has been introduced and seen rapid increased use: a recent UK-based survey and cohort study of SWHSI found an increase from 6 to 29% in a 1-year period [4].

NPWT was developed in the 1990s as a treatment for open wounds (although versions for closed wounds are now available they are not the focus here). The device applies a controlled negative pressure (vacuum) to a wound via a specialist dressing, removing wound fluid into a canister [9]. Kinetic Concepts Inc. (KCI), the manufacturer who pioneered the use of NPWT with their V.A.C® device, claim that the mechanical forces generated by the negative pressure create a wound environment that is conducive to healing by removing infective materials and exudate, reducing oedema and promoting perfusion and granulation [10]. NPWT is not anticipated to be used to the point of healing, rather its use is promoted as part of the treatment pathway to reduce the time taken for healing to be achieved.

Despite the increasing use of NPWT as a treatment for SWHSI, there is limited robust evidence to evaluate the effectiveness and cost-effectiveness of this treatment for this patient group. A Cochrane review identified three randomised controlled trials [11–14] of NPWT as a treatment for SWHSI; however, all were small and caution was advised when interpreting the findings [9, 15]. The clinical and cost-effectiveness and the harms and benefits of NPWT in the treatment of SWHSI therefore remain uncertain.

### Objectives {7}

To assess the clinical and cost-effectiveness of NPWT compared to usual care (no NPWT) in treating SWHSI. This trial will test the hypothesis that NPWT is superior to usual care in treating SWHSI, based on time to healing in days from randomisation. A detailed economic evaluation will be undertaken to compare the cost-effectiveness of NPWT to usual care to determine the most efficient provision for future care and resources.

### Trial design {8}

SWHSI-2 is a pragmatic, multi-centre, cross surgical specialty, two-arm, parallel group, randomised controlled, superiority trial. Participants will be randomised in a 1:1 ratio to the two treatments (NPWT and usual care).

Funded by the MRC PROMETHEUS Programme (MR/R013748/1), the trial includes two nested studies within a trial (SWATs) to assess the effectiveness of strategies to improve recruitment (infographic plus participant information sheet (PIS) vs PIS only) and retention (thank you card sent between follow-up time points vs no card).

## Methods: participants, interventions and outcomes

### Study setting {9}

Participants will be recruited from up to 25 enrolled sites (acute NHS hospitals, community NHS trusts and primary care centres) in the UK. A list of enrolled sites is available in Supplementary File 1.

### Eligibility criteria {10}

The trial will include adult patients with a SWHSI who meet all of the inclusion criteria and none of the exclusion criteria.

#### Inclusion criteria

1. Aged 16 years or over
2. Has an acute SWHSI (i.e. a wound left open as planned following surgery or a wound initially closed using sutures, clips or other closure methods and dehisced along the whole or part of its length, and of less than 6 weeks in duration), arising from any surgical specialty and occurring on any part of the body, deemed appropriate to receive either NPWT or wound dressing treatment.
3. Has a SWHSI that is considered ready for NPWT treatment (i.e. contains at least 80% viable tissue or has only a very thin layer of slough requiring no further debridement).
4. Patient is not deemed to be malnourished, as per NICE guidelines CG 32 (33) (BMI < 18.5 kg/m^2^; unplanned* weight loss > 10% in the last 3–6 months; BMI < 20 kg/m^2^ and unplanned* weight loss > 5% in the last 3–6 months) or assessed as at high risk of malnutrition using the Malnutrition Universal Screening Tool (MUST) (34).
  *Note: Patients with weight loss arising either from underlying comorbidity (e.g. ulcerative colitis) or from the reasons for surgery being completed (e.g. bowel cancer) may be included at the healthcare professional’s discretion.
5. Willing and able to give informed consent and provide follow-up data

#### Exclusion criteria

1. Life expectancy of less than 6 months, e.g. undergoing end stage palliative care
2. Active systemic infection (including osteomyelitis) at baseline as defined by clinical and/or laboratory assessment. Note: Patients who have an active infection, but are improving following 1 week’s duration of antibiotics, may be included at the healthcare professional’s discretion.
3. Inadequate haemostasis or patients who are at risk of bleeding
4. Chronic wounds non-surgical in origin (e.g. pressure ulcers or foot ulcers*)
  *Note: diabetic foot ulcers that have been incised and drained or debrided as an inpatient in theatre may be included given this constitutes a surgical wound
5. Current open wound has previously been, or is currently being, treated with NPWT.
6. Planned delayed primary closure of the wound
7. Contraindication to NPWT including the following:
  - Presence of unclear undermining in the wound cavity (i.e. the deepest point of the wound cannot be measured)
  - Presence of necrotic tissue, malignant tissue or eschar
  - Wounds involving exposed blood vessels and/or organs, anastomotic sites and/or nerves (including the “open abdomen” where the abdominal fascia is open)
  - Wounds situated where, in the opinion of the treating healthcare professional, a vacuum seal cannot be obtained
  - Presence of a non-enteric or unexplored fistula
  - People requiring emergency airway aspiration, pleural mediastinal or chest tube drainage or surgical suction
8. Currently participating in another wound research study, where the primary outcome has not yet been reached

### Who will take informed consent? {26a}

Screening to identify eligible patients for the trial will occur initially in the surgical departments of participating NHS hospitals, community NHS trusts or primary care settings. Patients with a potential planned SWHSI (pre-operatively) or a SWHSI occurring at any point following surgery will be screened for potential eligibility by their clinical care team.

Potential participants will be approached with further details of the trial, including an information sheet, during a ward round, routine care or home visit by the clinical care team or research nurse (subject to permission from the participant if not part of the clinical care team).

The information sheet will clearly state that participants are free to withdraw from the study at any time for any reason without prejudice to future care and with no obligation to give the reason for withdrawal. Should new information arise during the study which may affect an individual’s willingness to take part, this will be reviewed for addition to the patient information sheet and a revised consent form will be completed as necessary.

The participant will be allowed as much time as they wish to consider the information and will be given the opportunity to consult with the Principal Investigator, the research team, their GP or other independent parties to decide whether they will participate in the study.

Potential participants will then be shown the consent form and will be given the opportunity to ask questions about the study.

The participant must personally sign and date the latest approved version of the informed consent form before any study specific, baseline procedures are performed. Informed consent will be obtained by a suitably qualified and experienced local research nurse or healthcare professional who has been authorised to do so by the Chief or Principal Investigator.

Participants are not informed about the two nested sub studies included in this trial and therefore cannot provide their informed consent for their involvement. Due to the nature of the nested sub study interventions (infographic plus PIS vs PIS alone; thank you card vs no thank you card), this approach is deemed low risk.

### Additional consent provisions for collection and use of participant data and biological specimens {26b}

There are no biological specimens collected within the SWHSI-2 trial; therefore, additional consent for collection and use is not required.

#### Explanation for the choice of comparators {6b}

Comparators were selected as NPWT and usual care (no NPWT) given the SWHSI-2 primary objective is to assess the clinical and cost effectiveness of these for the treatment of SWHSI.

As the mechanical principles and actions of NPWT devices are similar and there is no evidence to suggest clinical or cost effectiveness differences between devices, use of any CE marked NPWT device, providing pressure of 60–150 mmHg, in use within the NHS will be permitted in this trial.

Similarly for the usual care arm, given there is no evidence to suggest any one dressing is more clinically and cost-effective than another [16], use of any non-NPWT dressing type will be permitted.

### Intervention description {11a}

#### Intervention (negative pressure wound therapy — NPWT)

Negative pressure wound therapy (NPWT) devices are used as part of the SWHSI treatment pathway rather than necessarily to the point of healing and are administered by a range of healthcare professionals.

Prior to application of NPWT, the wound is filled with a suitable NPWT-specific dressing (for example, black, polyurethane foam dressings with reticulated (open) pores; white, polyvinyl alcohol foam with high tensile strength, pre-moistened with sterile water; or antimicrobial gauze (impregnated with polyhexamethylene biguanide)). A liner may also be placed in the wound bed to prevent dressing adherence. A controlled negative pressure is then applied to the wound via a specialist vacuum pump dressing. The dressing is attached to tubing, which enables wound fluid to be removed into a canister. The canister is removed and changed weekly or when it becomes full, whichever occurs first.

The device will be used in accordance with manufacturer guidance. The clinical care team, in conjunction with local treatment guidelines, will determine the duration of device use, whether this includes continuous or intermittent pressure cycles, and the type of dressing used. Treatment regimen and details will be recorded during weekly telephone follow-up.

#### Control (usual care, no NPWT)

Usual care will be that which is used locally by the NHS Trust, without NPWT. Most aspects of wound care will be the same except for the type of dressing used. The clinical care team will determine the dressing type (primary and secondary) and frequency of dressing change. Treatment regimen and details will be recorded during weekly telephone follow-up.

### Criteria for discontinuing or modifying allocated interventions {11b}

Given the pragmatic nature of the trial, the decision for discontinuation of the intervention or control treatment will be made by the clinical care team in conjunction with the participant. Details of discontinuation and any alternative treatments provided will be recorded during weekly telephone follow-up.

During the study, modifications may be made to the treatment as required, for example the duration of NPWT device use, changes to continuous or intermittent pressure cycles and the type of dressing used (NPWT and usual care). Changes will be made by the clinical care team and details of any changes will be recorded during weekly telephone follow-up.

### Strategies to improve adherence to interventions {11c}

Decisions for continuation or discontinuation of interventions will be at the discretion of the clinical care team, in conjunction with the participant and so no specific strategies have been included to improve intervention adherence.

### Relevant concomitant care permitted or prohibited during the trial {11d}

Throughout the study, concomitant medications or treatments deemed necessary may be prescribed. Details of these medications or treatments will be recorded during weekly telephone follow-up.

### Provisions for post-trial care {30}

At the end of the trial, participants will return to the care of their treating healthcare professional to determine any further treatment required. This may or may not include NPWT or usual care dressings as appropriate.

### Outcomes {12}

#### Primary outcome

The primary outcome for SWHSI-2 is time to healing (defined using the clinical criterion “complete epithelial cover in the absence of a scab”) in days from randomisation.

Participants will be contacted weekly by a research nurse and asked if a healthcare professional has indicated that their wound has healed and whether this meets the healing definition (i.e. there is full coverage and no scab present). If the participant believes the wound is healed, but this has not been confirmed by a healthcare professional, the research nurse will seek confirmation/further information from the clinical team.

Once healing has been confirmed, participants will undergo clinical assessments on 3 subsequent consecutive weeks to confirm continued wound healing. A standardised photograph will be taken of the wound at the first healing assessment. Participants (with assistance from family/friends if necessary) will also be asked to take a digital photograph of the wound themselves and submit this to the study team. Study specific instructions will be provided to facilitate this.

Given the subjective nature of healing assessment (23), the photographs will be used to facilitate additional outcome (time to healing) verification by clinically experienced, independent, blinded observers.

#### Secondary outcomes

Secondary outcomes are as follows:

1. Clinical events including antibiotic treatment, hospital admission or discharge, treatment status (including reasons for dressing or treatment failure or change), re-operation (including skin grafting and closure surgery*), amputation and death. Details of any clinical events experienced will be collected during the weekly telephone follow-up.
2. Wound infection using a modified Bluebelle Wound Healing Questionnaire (WHQ) [17, 18]. This will be completed by the participant at baseline, 3-, 6- and 12-month follow-up assessments and at the initial healing visit. The WHQ will be modified for relevance to patients with open wounds by removing three items relating to spontaneous or deliberate wound dehiscence and use of dressings given these would not be relevant in this population. The time frame for the questionnaire will also be adapted to reflect the questionnaire should be completed since the participant had their open wound (baseline and wound healing, or since the last Bluebelle questionnaire was completed (months 3, 6 and 12) rather than from the time since hospital discharge.
3. Pain using a visual analogue scale (0 = no pain — 10 = worst imaginable pain). This will be completed by the participant at baseline, 3-, 6- and 12-month follow-up assessments.
4. Health-Related Quality of life using the EQ-5D-5L [19]. This will be collected at baseline and 3, 6 and 12 months post-randomisation.
5. Resource use, i.e. wound-related NHS consultations, support (e.g. occupational therapy, in home adaptations) and out-of-pocket costs, will be completed by the participant at baseline, 3, 6 and 12 months. Details of wound dressing changes (frequency and type) will be collected at weekly follow-up. Information on resources provided by the recruiting centre will be collected via a retrospective review of medical records at 12 months post-randomisation.

Additionally, data including demographics, comorbidities, smoking status, surgical procedure details (e.g. urgency, contamination level [20], type) and a wound photograph will be collected at baseline.

#### SWAT outcomes

The primary outcome of the recruitment infographic SWAT will be the recruitment rate, i.e. the proportion of participants in each group who are randomised into the host trial. Secondary outcomes will include the proportion of patients in each group who are screened but do not go on to be randomised, and the cost-effectiveness of the intervention.

For the retention thank you card SWAT, the primary outcome will be questionnaire response rate, i.e. the proportion of participants who return their completed questionnaires at month 6 and 12 follow-up in each group. Secondary outcomes will include whether a reminder notice is required, completeness of response and cost of the intervention per participant retained.

### Participant timeline {13}

**Fig. 1:**
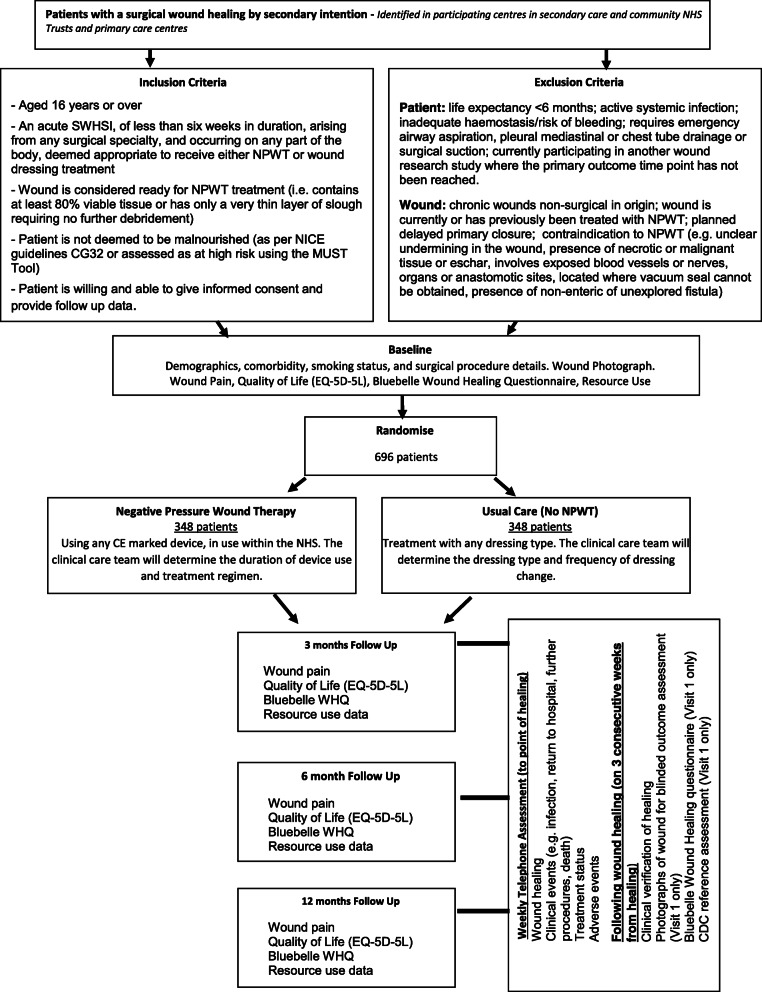
Study flow diagram

The study flow diagram is presented in Fig. 1.

### Sample size {14}

To detect a 25% reduction in median time to healing (from 86 days with usual care to 65 days with NPWT), with 90% power, 12-month follow-up, and allowing for 20% attrition [8, 13, 21], 696 participants are required to be recruited and randomised (348 NPWT; 348 usual care).

A conservative estimate of a 25% decrease in median time to healing is used to derive this sample size, assuming a median time to healing of 86 days in the usual care group, which equates to a 21-day reduction in time to healing to 65 days in the NPWT group.

The 25% reduction in time to healing was selected on the following basis:

- *Cost-effectiveness*. Models generated using observational data [8] suggest a 57.4% difference in time to healing would be required to demonstrate cost-effectiveness of NPWT. This should, however, be interpreted with caution given this relative effect is derived from observational data.
- *Current literature*. The average median time to healing in the control group of previous observational and randomised controlled trials is 86 days, with an average decrease in time to healing of 25% when NPWT is used [11–13, 22].
- *Significance to patients*. Patients are frequently disappointed by the slow healing process of a SWHSI and complete wound healing is therefore a major focus for patients [7]. Patient representatives have confirmed that the proposed reduction in time of 21 days with NPWT is likely to be significant for patients.

For the included recruitment and retention studies within a trial, as is usual with nested trials, a formal power calculation to determine sample size has not been conducted as the sample size is constrained by the number of patients approached about, or recruited into, the study, respectively.

### Recruitment {15}

Recruitment will be undertaken at NHS hospitals, community NHS Trusts and primary care centres. Participants can be recruited from any surgical specialties within these centres.

Participants with a planned SWHSI can be approached prior to surgery and those with unplanned SWHSIs or whose wound dehisces can be approached after surgery. Where patients are screened pre-operatively, consent will be obtained pre-operatively and randomisation completed either in theatre or post operatively. Baseline data will be collected post-operatively.

To supplement recruitment, a study within a trial (SWAT) testing an infographic to aid recruitment is nested within the SWHSI-2 trial. This SWAT will evaluate the effects of presentation of the study design to participants on recruitment rate. Participants will be cluster randomised (at the site level) to receive an infographic (visual document explaining the study) plus the standard PIS or just the PIS.

A recent review showed that tailoring or shortening the PIS given to participants makes little or no difference to recruitment [23]; however, infographics have been shown to improve patient knowledge and experience [24–26]. This suggests that infographics, in the context of health care research, may improve potential participants’ experience and understanding of health research thus leading to increased recruitment.

### Assignment of interventions: allocation

#### Sequence generation {16a}

Independent 1:1 random allocation to the two treatment arms will be used. This will be stratified by:

1. Wound location: foot and ankle, leg, abdomen or other
2. Wound area (< 28 cm^2^, ≥ 28 cm^2^)
3. Study centre

Stratification variables of wound location and wound area will be used as these have been found to impact on time to wound healing [8], whilst stratification by centre will be included to balance resource use across participating NHS Trusts. Variable blocks sizes will be used within strata.

For the recruitment SWAT, minimisation will be utilised to allocate sites based on the following factors: (i) whether the site is recruiting cross specialty or in a single specialty and (ii) expected number of eligible participants as reported on the site feasibility assessment cut at the median.

For the retention SWAT, participants will be allocated 1:1 using block randomisation stratified by host trial treatment arm, using randomly varying block sizes.

#### Concealment mechanism {16b}

Randomisation for the main trial will be completed by a centralised secure randomisation service hosted by York Trials Unit, University of York. Randomisation will be completed via the internet, with information recorded to check eligibility prior to randomisation.

#### Implementation {16c}

The allocation sequence for the main trial will be generated by the trial statistician who is independent to the recruiting teams at participating NHS Trust sites. This sequence will be implemented using the secure randomisation service that can be accessed by staff recruiting participants and will assign participants to either NPWT or standard care.

Similarly for the nested recruitment and retention SWATs, generation of the allocation sequence will be undertaken independently by a researcher not involved with the recruitment of participants.

### Assignment of interventions: blinding

#### Who will be blinded {17a}

As the study treatments cannot be adequately concealed, neither the trial participants nor the clinical care or research teams will be blinded to treatment allocation.

To limit potential bias in relation to primary outcome assessment, photographs taken of the wound following healing will be used to facilitate additional outcome (time to healing) verification by clinically experienced, independent, blinded observers.

For the two nested sub studies, as participants will not be informed of these methodological studies they will not be able to provide informed consent for their involvement and will therefore be blinded to their embedded trial allocation.

#### Procedure for unblinding if needed {17b}

As study treatments cannot be blinded, there is no requirement for an unblinding procedure in this study.

Given the low risk of the nested sub studies, there is no requirement for an unblinding procedure for these components either.

### Data collection and management

#### Plans for assessment and collection of outcomes {18a}

Data will be collected at baseline and at 3, 6 and 12 months post-randomisation, and via weekly clinical telephone follow-ups.

At baseline, epidemiological data will be collected including patient demographics, comorbidities, smoking status and surgical procedure details. Participants will complete a questionnaire comprising of a number of outcomes measures including surgical site infection (Bluebelle WHQ [17]), wound pain (using a visual analogue scale), quality of life (EuroQol EQ-5D-5L [19]) and resource use (using a bespoke, study specific questionnaire, which was designed based on the experiences learnt from the earlier feasibility study [27]).

At 3, 6 and 12 months, participants will be sent a postal questionnaire to complete and return comprising of a number of outcomes measures including surgical site infection (Bluebelle WHQ [17]), wound pain (using a visual analogue scale), health-related quality of life (EuroQol EQ-5D-5L [19]) and resource use (using a bespoke, study specific questionnaire). Where no response is provided after 2 weeks, a reminder questionnaire will be sent. If after 2 further weeks no response is received, a reminder telephone call will be made to the participant, and data collected by telephone if the participant is agreeable.

During the trial, participants will also be telephoned weekly to collect data on wound healing (using the criteria complete epithelial cover in the absence of a scab (eschar)), clinical events, treatment status and adverse events. When the participant reports that their treating healthcare professional has indicated that their wound is healed, and this has been confirmed in accordance with the SWHSI-2 trial definition (as above), the research nurse will undertake an assessment with the participant, ideally within 48 h of the report of healing being made, to confirm that the wound has healed. In the event wound healing has not been confirmed by a healthcare professional, the research nurse will first contact the clinical care team to obtain this confirmation and the date the participant was advised their wound was healed will be taken as the date of wound healing.

When the wound is confirmed to be healed, clinical assessments will be completed with the participant on 3 consecutive weeks (commencing with the initial healing visit). At the initial visit a standardised photograph will be taken to facilitate blinded outcome verification, and the Bluebelle WHQ [17] will be completed. The research nurse will also determine whether surgical site infection (SSI) has occurred in the time since the participant has had an open surgical wound using a SSI reference assessment (Centre for Disease Control (CDC) criteria, assessed retrospectively. This CDC assessment is required as a reference assessment to enable validation of an SSI threshold score for the Bluebelle WHQ in patients with open surgical wounds.

Wherever possible the initial visit will be completed face to face at the participant’s home or in a clinical care setting to facilitate provision of the standardised photograph taken by the research nurse. Where this is not possible, a video call, using NHS approved technologies, will be arranged and a photograph taken via screen shot. If this is also not possible, the participant will be contacted by telephone and will be asked to take and to return (by electronic submission) a photograph of their wound. Subsequent healing visits (weeks 2 and 3) may then be completed by telephone, if preferred, to confirm continued wound healing.

At 12 months following randomisation, the research team will conduct a review of participants’ NHS medical records to identify and document any resources used, in relation to the participants wound, during the preceding 12 month period.

#### Plans to promote participant retention and complete follow-up {18b}

With the study primary outcome being time to wound healing, if participants wish to withdraw involvement from the trial, we will seek, where possible, consent to obtain data on healing status from the participant’s treating healthcare professional. This should ensure attrition of the primary outcome data is limited.

To ensure we are able to collect as much participant reported questionnaire data as possible, a range of strategies will be employed. Firstly, if there is no response to a postal questionnaire after 2 weeks, a reminder letter and questionnaire will be sent. If there is still no response after a further 2 weeks, the participant will be telephoned to collect their data. In addition, if for any reason a questionnaire cannot be posted to the participant, the study team may contact the participant by telephone in order to complete data collection. Secondly, participants will be sent a pen with each follow-up questionnaire and will receive an unconditional £5 with both the 6- and 12-month questionnaires. Both strategies have been found to have an effect in improving participant retention and questionnaire response rates [28–30]. Participants will also be sent a study newsletter 2 weeks before the 6-month follow-up time point to encourage continued engagement.

A SWAT testing a thank you card is also embedded within the SWHSI-2 trial. Participants will be individually randomised to either receive a thank you card at months four and nine following recruitment or to receive no thank you card at these time points.

Many recruitment and retention strategies routinely include some element of thanks within them; however, there is little to no evidence to suggest these work. There is therefore a need to test the impact of thanks in the context of trial retention.

#### Data management {19}

Participant data as required by the protocol will be recorded on case report forms (CRF). These will be completed at baseline, treatment delivery, weekly assessment, post wound healing and at 3, 6 and 12 months post-randomisation. Separate CRFs will be used to collect clinical information and patient reported information. Copies of the CRFs used in the trial are available as supplementary files.

To ensure high quality data, data collected within the CRFs will be processed at the York Trials Unit (YTU, University of York), using a licensed, automated, electronic system (Teleform), which allows data to be entered, checked and validated. Further details pertaining to the processing of the data will be documented in a study specific data management plan.

Study documentation will be retained in accordance with Good Research Practice and UK Law for five years after study completion in the Trial Master and Investigator Site Files, after which time information will be securely destroyed.

#### Confidentiality {27}

To ensure confidentiality, all participants will be allocated a unique coded ID number, which will be used to identify them throughout the trial.

Personal data held electronically will be stored on a data management system which will be accessible to delegated members of the coordinating team via individual password. Paper forms containing participant identifiable information (e.g. patient details form and consent form) will be held separately to questionnaire data in a locked filing cabinet, in an office only accessible via registered swipe card access held by the YTU research team. Photographs to record wound healing will be anonymised prior to electronic transfer by sites to YTU, where the image will be stored in an encrypted and password protected drive. All data will be stored and handled in accordance with data protection principles.

To ensure that the study is carried out correctly, participants’ data may be reviewed by members of the research team or other authorised people, maintaining confidentiality at all times. Participants will consent to this review of their data at the start of the study. Identifying information will be removed before the data is analysed and the results presented and published.

#### Plans for collection, laboratory evaluation and storage of biological specimens for genetic or molecular analysis in this trial/future use {33}

There will be no biological specimens collected within the SWHSI-2 trial; therefore, no plans are required for collection, evaluation or storage of such specimens.

### Statistical methods

#### Statistical methods for primary and secondary outcomes and additional analyses {20a, 20b}

##### Statistical analysis

Outcome analyses will be conducted following the principles of intention-to-treat with participant’s outcomes analysed according to their original, randomised group. Analysis will be undertaken using Stata version 16 or later (College Station, TX: StataCorp LLC). Significance tests will be two-sided at the 5% significance level, and parameter estimates will be presented with associated 95% CI and *p*-values as appropriate.

Baseline data will be summarised by randomised group, as randomised and as included in the primary analysis [31], using descriptive statistics for continuous variables and count and percentage for categorical variables. No formal statistical comparison of baseline data will be made between the groups.

For the primary outcome of time to healing of the reference wound (derived as the difference in days between randomisation and the first date of complete healing), healing rates will be presented overall and by trial arm. Kaplan–Meier survival curves will be produced for the two groups and the median time to healing with a 95% CI presented. A proportional hazards Cox regression model will be used to compare the healing times between the two groups, adjusting for wound size at baseline, duration of wound in days (time between wound start date and randomisation), and wound location as fixed effects, and centre as a shared frailty effect.

Secondary outcomes, including adverse events, will be analysed where appropriate using regression techniques appropriate for the type of data.

Full details for the statistical analysis will be provided in a statistical analysis plan, which will be prepared prior to the completion of data collection.

#### Methods in analysis to handle protocol non-adherence and any statistical methods to handle missing data {20c}

To assess the impact of compliance on the primary outcome we will consider a complier average causal effect (CACE) analysis. This will produce an unbiased estimate of the treatment effect in the presence of non-compliance (defined as participants in the NPWT group who do not receive NPWT). Participants in the standard care group who receive NPWT will be considered as a cross-over.

For all outcomes, the level of missing data and the missing data mechanism will be assessed, and if required appropriate imputation techniques will be considered.

##### Economic analysis

The economic analysis will aim to evaluate the lifetime cost-effectiveness of NPWT, compared to all relevant comparators, in the treatment of SWHSI. The perspective of the cost-effectiveness analysis will be of the UK NHS and the Personal Social Services. The primary economic outcome will be the incremental cost-effectiveness ratio for NPWT vs usual care, expressed as cost per Quality Adjusted Life Years (QALYs) gained.

In addition, and to best assist decision-making, we will aim to build a de novo decision analytic model to establish which treatment is cost-effective, under current information. Evidence from the SWHSI-2 trial will be key to inform parameters of this economic model and the wider existing evidence base, identified through literature reviews, will also be used, including evidence from our previous cohort study [8]. When required and suitable, evidence synthesis techniques will be used to pool data to best inform specific decision model input parameters.

Regression approaches will be used to derive costs and health benefits (health utility measured using EQ-5D-5L), allowing for correlation between these as well as adjusting for key covariates. Alternative scenarios regarding the extrapolation of the primary outcome over the lifetime of the model, and the evidence informing it, will be explored. Uncertainty in the evidence base used to populate the decision analytic model will be characterised using appropriate distributions and uncertainty in the adoption decision will be demonstrated using probabilistic sensitivity analysis. The value of further data collection will be established using a value of information analysis.

Full details of the health economic analysis will be provided in a health economic analysis plan, which will be prepared prior to the completion of data collection.

##### SWAT analyses

For both SWATs, logistic regression will be used to assess the difference in binary outcomes, e.g. recruitment and retention rates. Factors used in the minimisation will be included as fixed effects in the analysis models for in the recruitment SWAT, with main trial allocation being adjusted for in the retention SWAT. For all analyses, site will be included as a random effect.

The difference in costs per recruited and retained participants will be calculated, including direct and indirect costs where applicable.

#### Interim analyses {21b}

There are no planned interim analyses and no planned stopping rules for this trial.

### Plans to give access to the full protocol, participant level-data and statistical code {31c}

This document constitutes the full protocol. Datasets and statistical code used in this study will be available from the corresponding author on reasonable request following completion of the trial.

### Oversight and monitoring

#### Composition of the coordinating Centre and trial steering committee {5d}

The coordinating team will comprise the sponsor and Chief Investigator (based at Hull University Hospitals NHS Trust) and the trial manager, trial coordinators, data management and administrative support (based at York Trials Unit, University of York). The coordinating team will ensure all relevant approvals are in place, will train and support sites to undertake the study and will put measures in place to obtain accurate data. The data management team will process and check data against validation criteria agreed with the trial manager.

The trial management group (TMG) will comprise patient representatives, the sponsor, Chief Investigator, trial manager, trial statisticians, health economists, trial coordinators, co-applicants (clinicians, nurses, researchers) and administrative support staff. The TMG will meet every 2 months to review trial progress and safety.

The Trial Steering Committee (TSC) will comprise an independent chair, three independent members with clinical expertise and two patient representatives. The TSC will meet at least annually to provide overall supervision for the trial on behalf of the sponsor and funder.

#### Composition of the data monitoring committee, its role and reporting structure {21a}

The data monitoring committee (DMC) will comprise a clinician, statistician and nurse, independent of the project team or any institution recruiting for this study.

The DMC will undertake ongoing review of the SWHSI-2 trial’s progress, assessing recruitment, data quality, outcome/efficacy data and safety data. The DMC will meet at least once a year and will make written recommendations with regards continuation of the trial to the TSC.

### Adverse event reporting and harms {22}

For the SWHSI-2 trial, adverse events (AE) will be defined as any untoward medical occurrence experienced by a clinical trial participant, which is temporally associated with study treatment (interventions or control) and is related to the wound or to the study intervention or control treatments.

Adverse events, which might be expected with these wounds, include minor wound infection, cellulitis, oedema, maceration and retention of product in the wound, e.g. wound filler embedding in granulated tissue.

Serious adverse events (SAE) will be defined as any untoward medical occurrence:

1. Results in death
2. Is life threatening
3. Requires unplanned inpatient hospitalisation or prolongation of existing inpatients’ hospitalisation
4. Results in persistent or significant disability or incapacity
5. Is a congenital anomaly or birth defect
6. Any other important medical condition that, although not included in the above, may require medical or surgical intervention to prevent one of the outcomes listed

For the purposes of the SWHSI-2 trial, hospitalisation for the treatment of major wound infection, osteomyelitis, wound bleeding, fistulation, for removal of embedded wound filler and for limb amputation, will not be considered a SAE but will be considered and reported as an AE.

Where an adverse event is reported, participating sites will be required to promptly report this to YTU. Where the event is considered serious, causality and expectedness will be confirmed by the Chief Investigator or another clinical member of the SWHSI-2 management team. Where events are unexpected and related these will be reported to the research ethics committee and Sponsor within 15 days.

Adverse and serious adverse events will be routinely reported to the DMC and TSC for their review and oversight.

### Frequency and plans for auditing trial conduct {23}

No on site auditing of trial conduct will be completed, unless circumstances prevail (e.g. serious breach of GCP) that warrant this. Centralised monitoring checks of eligibility and consent will be completed for 100% of participants and an annual audit of site files and documentation, via a self-complete checklist, will be completed with each participating NHS Trust. Further details relating to these activities will be documented in a study specific monitoring plan.

The TMG will meet very two months to continuously evaluate the conduct of the study, in addition to routine review by the independent DMC and TSC.

### Plans for communicating important protocol amendments to relevant parties (e.g. trial participants, ethical committees) {25}

Any protocol amendments will be approved by the Sponsor (Hull University Hospitals NHS Trust) and the Funder (NIHR Health Technology Assessment Programme) prior to submission to the approving Research Ethics Committee (Yorkshire and Humber Leeds East) and the Health Research Authority. Documentation will be provided to study sites for their local review and implementation as required.

### Dissemination plans {31a}

Following completion of the SWHSI-2 Trial, irrespective of the magnitude of effect, we will submit the study results to peer reviewed journals. The executive summary and trial report will also be sent to the National Institute for Health and Care Excellence (NICE) and other relevant bodies, including Clinical Commissioning Groups, to facilitate translation into clinical practice.

We will produce a plain English summary of the report and disseminate this to participants, members of our patient advisory group and relevant patient-focused websites. Patient information will also be generated for “Shared Decision Making”, the entry on Wikipedia and the Map of Medicine entry.

## Discussion

Use of NPWT as a treatment for SWHSI has been rapidly increasing despite there being limited high-quality evidence to support its clinical and cost-effectiveness. Given the increasing use of this device in routine care, a full and sufficiently powered randomised controlled trial is essential to evaluate the effectiveness of this treatment for SWHSI. The SWHSI-2 trial is therefore designed to generate robust evidence to fill this gap.

### Study status

Recruitment to the SWHSI-2 Trial commenced in May 2019, and to date 252 participants of the target 696 (36.2%) have been recruited. Twenty sites are currently open to study recruitment, with recruitment of at least one participant having occurred at 18 sites to date.

The impacts of the COVID-19 pandemic resulted in a pause of recruitment between 23 March and 29 July 2020. Subsequent waves of COVID-19, including the presence of new variants across the UK, have limited the study’s ability to recruit to target due to substantial reductions in elective surgeries across the NHS, due to staff sickness and/or isolation, and the requirement of NHS Trusts to prioritise urgent public health studies in relation to COVID-19. Recruitment activity will therefore continue to ensure sufficient recruitment to enable a powered analysis.

Despite COVID-19, attrition remains low within the study. Use of a clinical, rather than patient-reported, primary outcome (time to wound healing in days since randomisation) has enabled collection of primary outcome data from treating healthcare professionals, if required and subject to participant consent, which has reduced any potential attrition for this key outcome. Follow-up assessment of participants through use of telephone and video assessments, conducted by the coordinating team when required, has been used throughout to minimise attrition for secondary outcomes.

Results of the SWHSI-2 study are currently expected in 2022.

## Supplementary Information

**Additional file 1.** Enrolled SWHSI-2 Study Sites

**Additional file 2.** One Year Participant Questionnaire Booklet

**Additional file 3.** Weekly Assessments - Monthly Case Report Form

**Additional file 4.** Treatment Delivery Case Report Form

**Additional file 5.** SWHSI-2 Supplementary Page for Outpatient Visits

**Additional file 6.** SWHSI-2 Supplementary Page for Inpatient Admissions

**Additional file 7.** SWHSI-2 Supplementary Page for Accident and Emergency Visits

**Additional file 8.** SWHSI-2 Supplementary Page for Infection CDC Assessment

**Additional file 9.** Post Healing Assessment Case Report Form

**Additional file 10.** Participant 6 Month Questionnaire Booklet

**Additional file 11.** Participant 3 Month Questionnaire Booklet

**Additional file 12.** 12 Month Resource Use - Case Report Form

**Additional file 13.** Participant Baseline Questionnaire Booklet

**Additional file 14.** Baseline Investigator Case Report Form

**Additional file 15.** Screening for Eligibility Case Report Form

**Additional file 16.** SWHSI-2 Participant Change of Status Form

**Additional file 17.** SWHSI-2 Blinded Outcome Assessment CRF

**Additional file 18.** Participant Information Sheet

**Additional file 19.** PARTICIPANT CONSENT FORM

## Abbreviations

AE: Adverse event
BMI: Body mass index
CACE: Complier average causal effect
CDC: Centre for Disease Control
CI: Confidence interval
CRF: Case report form
DMC: Data monitoring committee
EQ-5D-5L: EuroQol 5 Domain 5 Level
GCP: Good clinical practice
ID: Identification
KCI: Kinetic Concepts Inc.
MRC: Medical Research Council
MUST: Malnutritional universal screening tool
NHS: National Health Service
NICE: National Institute for Health and Care Excellence
NIHR: National Institute for Health Research
NPWT: Negative pressure wound therapy
PIS: Participant information sheet
QALY: Quality adjusted life year
SAE: Serious adverse event
SWAT: Study within a trial
SWHSI: Surgical wound healing by secondary intention
TMG: Trial management group
TSC: Trial steering committee
WHQ: Wound Healing Questionnaire
YTU: York Trials Unit
