# Supplementary material for: Negative pressure wound therapy versus usual care for surgical wounds healing by secondary intention (SWHSI-2 trial): study protocol for a pragmatic, multicentre, cross surgical specialty, randomised controlled trial
Source: Trials. 2021 Oct 25;22:739. doi: 10.1186/s13063-021-05662-2 (PMC8543414; doi:10.1186/s13063-021-05662-2)
Supplement: Supplementary file 1 — Additional file 1. Enrolled SWHSI-2 Study Sites [file 13063_2021_5662_MOESM1_ESM.docx]

**Supplementary File 1**

Enrolled SWHSI-2 Study Sites

| **Open and Recruiting** | |
| --- | --- |
| Hull University Teaching Hospitals NHS Trust | The Pennine Acute Hospital NHS Trust |
| University Hospitals of Birmingham NHS Foundation Trust (Queen Elizabeth Hospital) | St George’s University Hospital NHS Foundation Trust |
| University Hospitals of Birmingham NHS Foundation Trust (Heartlands Hospital) | The Mid Yorkshire Hospitals NHS Trust |
| Imperial College Healthcare NHS Trust | Northumbria Healthcare NHS Foundation Trust |
| The Dudley Group NHS Foundation Trust | Leeds Teaching Hospitals NHS Trust |
| Doncaster and Bassetlaw Hospitals NHS Foundation Trust | The Queen Elizabeth Hospital Kings Lynn NHS Foundation Trust |
| Norfolk and Norwich University Hospitals NHS Foundation Trust | Aneurin Bevan University Health Board |
| Royal Cornwall Hospitals NHS Trust | University Hospitals of Derby and Burton |
| The Newcastle Upon Tyne Hospitals NHS Foundation Trust | South Tyneside and Sunderland NHS Foundation Trust |
| NHS Lanarkshire |  |
| **In Active Set Up** | |
| Bradford Teaching Hospitals NHS Foundation Trust | NHS Lothian |
