# Supplementary material for: Negative pressure wound therapy versus usual care for surgical wounds healing by secondary intention (SWHSI-2 trial): study protocol for a pragmatic, multicentre, cross surgical specialty, randomised controlled trial
Source: Trials. 2021 Oct 25;22:739. doi: 10.1186/s13063-021-05662-2 (PMC8543414; doi:10.1186/s13063-021-05662-2)
Supplement: Supplementary file 3 — Additional file 3. Weekly Assessments - Monthly Case Report Form [file 13063_2021_5662_MOESM3_ESM.pdf]

The Department of Health Sciences

SWHSI-2 Trial

Weekly Assessments - Monthly Case Report Form

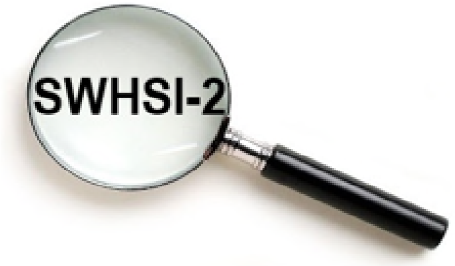

Site ID: ☐☐

Participant ID: ☐☐☐☐

This project is funded by the National Institute for Health Research Health Technology Assessment Programme (Project number 17/42/94)

Instructions for Completion

An entry should be made in this CRF for every ***completed*** *visit.*

This CRF may be completed by the principal investigator or a delegated member of staff listed on the SWHSI-2 Trial Delegation Log. There are sufficient spaces to record four participant assessments.

Please refer to the associated SWHSI-2 Trial Specific Procedure for full details of how to complete this CRF.

Please complete all sections of this CRF using the spaces provided, and sign off when complete.

Please do not include any patient identifiable information when completing this CRF. When complete, please remove the staple and take a photocopy of the completed CRF for your site records. ***Please do not re-staple the original.*** *Place the unstapled original in a "SWHSI-2 Trial business reply envelope" and send via post to York Trials Unit.*

Participant ID: | | | | | **Visit Date:** | | |/| | |/| | | | | (day / month / year)

Section A – Clinical Events

Please review any previous assessments and AE/SAE forms for ongoing events before completing this section.

Have any of the following events occurred since the last assessment?

Section A1 – Wound Healing

Event	Yes	No	Event Date (dd/mm/yyyy)
1) Has healing of the reference SWHSI (confirmed by a healthcare professional) occurred since the last visit?	☐	☐	__ __ / __ __ / __ __ __ __
a) If 'No', does the participant think their wound has healed?	☐	☐	

Section A2 – Clinical Events

Event	Yes	No	Event Date (dd/mm/yyyy)
1) Did the participant have an infected SWHSI at the last assessment?	☐	☐	
a) If 'Yes', has the infection resolved?	☐	☐	Date of Infection Resolution: __ __ / __ __ / __ __ __ __
2) Has the participant had a new SWHSI infection since their last assessment?	☐	☐	Date of New Infection: __ __ / __ __ / __ __ __ __
If 'Yes'... a) Has a SWHSI-2 Trial Adverse Event form been completed?	☐	☐	Adverse Event Number __ __
b) Has the new infection been resolved?	☐	☐	Date of Infection Resolution: __ __ / __ __ / __ __ __ __
3) Was the patient an inpatient in relation to their SWHSI or SWHSI treatment at the last assessment	☐	☐	
a) If 'Yes', has the participant been discharged?	☐	☐	Date of Discharge: __ __ / __ __ / __ __ __ __
4) Has the participant been admitted as an inpatient in relation to their SWHSI or SWHSI treatment since the last assessment?	☐	☐	Date of Admission: __ __ / __ __ / __ __ __ __
If 'Yes'... a) Has a SWHSI-2 Trial Adverse Event form been completed?	☐	☐	Adverse Event Number __ __
b) Has the participant been discharged?	☐	☐	Date of Discharge: __ __ / __ __ / __ __ __ __

5) Return to theatre due to SWHSI ☐ Yes ☐ No __ __ / __ __ / __ __ __ __ (dd / mm / yyyy)

a) If 'Yes', in theatre was the participant's SWHSI *(cross all that apply)*:

☐ Surgically closed (flap) ☐ Surgically closed (skin graft)
☐ Surgically closed (delayed primary healing) ☐ Debrided ☐ Revascularised
☐ Incised and drained ☐ Examined under anaesthetic ☐ Amputated
☐ Angioplasty ☐ Other (please specify) ________

b) If 'Yes', has a SWHSI-2 Trial Adverse Event form been completed? ☐ Yes ☐ No Adverse Event Number __ __

c) Where the participant's SWHSI was closed, was the decision for closure made by a clinician blind to treatment allocation? ☐ Yes ☐ No

d) If 'No', please indicate why this assessment was not blinded: ________

Please proceed to Section B

Participant ID: ☐☐☐☐

Section B – Treatment Status (Visit 1 only)

1. Where is the participant currently receiving treatment? *(cross all that apply)*

☐ Community Clinic ☐ Nursing Home ☐ Patient's home ☐ General ward ☐ ICU

☐ High Dependency Unit ☐ Other (please specify): ______________________

2. Has the participant's treatment location changed **since the last assessment**? ☐ Yes ☐ No

a) If 'Yes', please select where the treatment was being received previously *(cross all that apply):*

☐ Community Clinic ☐ Nursing Home ☐ Patient's home ☐ General ward ☐ ICU

☐ High Dependency Unit ☐ Other (please specify): ______________________

3. Please indicate the number of dressing changes the participant has received **since the last assessment** *(select one option)*:

☐ 1 ☐ 2 ☐ 3 ☐ 4 ☐ 5 ☐ 6 ☐ 7 ☐ 8 ☐ 9 ☐ 10

☐ 11 ☐ 12 ☐ 13 ☐ 14 ☐ Other (please specify): ☐☐

4. Please indicate the participants current treatment status *(select one option)*:

a) ☐ Patient continues to receive NPWT with no changes **since the last assessment**
If 'Yes', proceed to Section E

b) ☐ Patient continues to receive Usual Care with no changes **since the last assessment**
If 'Yes', proceed to Section E

c) ☐ Patient continues to receive NPWT but changes have been made **since the last assessment**
If 'Yes', proceed to Question 5

d) ☐ Patient continues to receive Usual Care but changes have been made **since the last assessment**
If 'Yes', proceed to Question 5

e) ☐ Patient has changed from NPWT to Usual Care **since the last assessment**
If 'Yes', proceed to Question 5

f) ☐ Patient has changed from Usual Care to NPWT **since the last assessment**
If 'Yes', proceed to Question 5

5. Please indicate why the patient has had changes made since the last assessment *(cross all that apply)*:

☐ Wound improving ☐ Wound healed ☐ Wound bed prepared

☐ Wound too dry ☐ Deterioration of wound ☐ Failure to maintain seal

☐ Treatment caused pain ☐ No change to wound

☐ Other (please specify): ______________________

Was the change at: ☐ Patient request ☐ Clinician request

If participant is receiving NPWT, proceed to Section C

If participant is receiving Usual Care, proceed to Section D

Participant ID: ☐☐☐☐

Section C – Changes to NPWT Treatment Please only complete if you have selected 4c or 4f

Please provide date change made: ☐☐ / ☐☐ / ☐☐☐☐
day month year

Please provide details of the NPWT treatment the patient is now receiving *(select one option)*:

1. ☐ V.A.C Ulta (KCI) ☐ V.A.C Via (KCI)
   ☐ InfoV.A.C (KCI) ☐ ActiV.A.C (KCI)
   ☐ Renasys (Smith and Nephew) ☐ PICO (Smith and Nephew)
   ☐ Avance (Molnlycke Health Care) ☐ Avance Solo (Molnlycke Health Care)
   ☐ Avelle (Convatec)
   ☐ Other (please specify model and brand): ________

2. Amount of pressure being used: ☐☐☐ *mmHg*
3. Is the pressure being applied: ☐ Continuously ☐ Intermittently
4. Type of NPWT dressing being used:
   ☐ Black, polyurethane foam dressings with reticulated (open) pores
   ☐ White, polyvinyl alcohol with high tensile strength, pre-moistened with sterile water
   ☐ Antimicrobial gauze (impregnated with Polyhexamethylene biguanide)
5. Has a liner been used with the dressing? ☐ Yes ☐ No

a) If 'Yes', was the liner silver impregnated? ☐ Yes ☐ No

Please proceed to Section E

Section D – Changes to Usual Care Treatment Please only complete if you have selected 4d or 4e

Please provide date change made: ☐☐ / ☐☐ / ☐☐☐☐
day month year

Please provide details of the Usual Care treatment the patient is now receiving *(cross all that apply)*:

☐ Alginate ☐ Basic wound contact
☐ Cavity Foam ☐ Foam
☐ Gauze Ribbon ☐ Hydrocolloid
☐ Hydrogel ☐ Hydrofoam/Spun Hydrocolloid
☐ Honey ☐ Iodine Containing
☐ PHMB ☐ Protease Modulating matrix
☐ Silver containing ☐ Flamazine silver sulfadiazine dressing
☐ Urgotol silver sulfadiazine dressing ☐ Soft polymer
☐ Superabsorbent ☐ Vapour permeable film or membrane
☐ Other (please specify): ________
☐ Other (please specify): ________

Please proceed to Section E

Section E – Other Treatments

1. Is the participant currently using antibiotics? ☐ Yes ☐ No

a) If 'Yes', please indicate the route of administration:

☐ IV ☐ Oral ☐ Other (please specify): ____________________

b) If 'Yes', please indicate the reason:

☐ Related to SWHSI ☐ Other (please specify): ____________________

2. Is the participant receiving any other treatment for their SWHSI? ☐ Yes ☐ No

a) If 'Yes', please provide details below *(cross all that apply)*:

☐ Wound Management Systems ☐ Emollient ☐ Silver Nitrate Stick

☐ Dietetic Input ☐ Larvae

☐ Other (please specify): ____________________

☐ Other (please specify): ____________________

☐ Other (please specify): ____________________

Please proceed to Section F

Section F – Visit Checklist and Sign Off

1. All Sections of the Weekly Assessment are complete? ☐ Yes ☐ No

2. Has the next weekly assessment (or first post wound healing visit if wound has healed) been arranged? ☐ Yes ☐ No

Form Completed by (to be completed by delegated clinician or nurse who has reviewed CRF content for this visit)

*Name: ____________________

Signature: ____________________

Assessor ID: ☐☐

Date: ☐☐ / ☐☐ / ☐☐☐☐
day month year

*Must be reflected in the Delegation of Duties Log

Participant ID: ☐☐☐☐ **Visit Date:** ☐☐ / ☐☐ / ☐☐☐☐
day *month* *year*

Section A – Clinical Events

Please review any previous assessments and AE/SAE forms for ongoing events before completing this section.

Have any of the following events occurred since the last assessment?

Section A1 – Wound Healing

**Event**	**Yes**	**No**	**Event Date** *(dd/mm/yyyy)*
1) Has healing of the reference SWHSI (confirmed by a healthcare professional) occurred since the last visit?	☐	☐	☐☐ / ☐☐ / ☐☐☐☐
a) If 'No', does the participant think their wound has healed?	☐	☐	

Section A2 – Clinical Events

**Event**	**Yes**	**No**	**Event Date** *(dd/mm/yyyy)*
1) Did the participant have an infected SWHSI at the last assessment?	☐	☐	
a) If 'Yes', has the infection resolved?	☐	☐	Date of Infection Resolution: ☐☐ / ☐☐ / ☐☐☐☐
2) Has the participant had a new SWHSI infection since their last assessment?	☐	☐	Date of New Infection: ☐☐ / ☐☐ / ☐☐☐☐
If 'Yes'... a) Has a SWHSI-2 Trial Adverse Event form been completed?	☐	☐	Adverse Event Number ☐☐
b) Has the new infection been resolved?	☐	☐	Date of Infection Resolution: ☐☐ / ☐☐ / ☐☐☐☐
3) Was the patient an inpatient in relation to their SWHSI or SWHSI treatment at the last assessment	☐	☐	
a) If 'Yes', has the participant been discharged?	☐	☐	Date of Discharge: ☐☐ / ☐☐ / ☐☐☐☐
4) Has the participant been admitted as an inpatient in relation to their SWHSI or SWHSI treatment since the last assessment?	☐	☐	Date of Admission: ☐☐ / ☐☐ / ☐☐☐☐
If 'Yes'... a) Has a SWHSI-2 Trial Adverse Event form been completed?	☐	☐	Adverse Event Number ☐☐
b) Has the participant been discharged?	☐	☐	Date of Discharge: ☐☐ / ☐☐ / ☐☐☐☐

5) Return to theatre due to SWHSI ☐ Yes ☐ No ☐☐ / ☐☐ / ☐☐☐☐ (*dd* *mm* *yyyy*)

a) If 'Yes', in theatre was the participant's SWHSI *(cross all that apply)*:

- ☐ Surgically closed (flap)
- ☐ Surgically closed (skin graft)
- ☐ Surgically closed (delayed primary healing)
- ☐ Debrided
- ☐ Revascularised
- ☐ Incised and drained
- ☐ Examined under anaesthetic
- ☐ Amputated
- ☐ Angioplasty
- ☐ Other (please specify) ____________

b) If 'Yes', has a SWHSI-2 Trial Adverse Event form been completed? ☐ Yes ☐ No Adverse Event Number ☐☐

c) Where the participant's SWHSI was closed, was the decision for closure made by a clinician blind to treatment allocation? ☐ Yes ☐ No

d) If 'No', please indicate why this assessment was not blinded: ____________

Please proceed to Section B

Section B – Treatment Status (Visits 2-4 only)

1. Has the location of the patient's treatment changed **since the last assessment**? ☐ Yes ☐ No

a) If 'Yes', please select where the patient is currently receiving treatment *(cross all that apply)*:

☐ Community Clinic ☐ Nursing Home ☐ Patient's home

☐ General ward ☐ ICU ☐ High Dependency Unit

☐ Other (please specify): ____________________

2. Please indicate the number of dressing changes the participant has received **since the last assessment** *(select one option)*:

☐ 1 ☐ 2 ☐ 3 ☐ 4 ☐ 5 ☐ 6 ☐ 7 ☐ 8 ☐ 9 ☐ 10

☐ 11 ☐ 12 ☐ 13 ☐ 14 ☐ Other (please specify): ☐☐

3. Please indicate the participants current treatment status *(select one option)*:

☐ Patient continues to receive NPWT with no changes **since the last assessment**
If 'Yes', proceed to Section E

☐ Patient continues to receive Usual Care with no changes **since the last assessment**
If 'Yes', proceed to Section E

☐ Patient continues to receive NPWT but changes have been made **since the last assessment**
If 'Yes', proceed to Question 4

☐ Patient continues to receive Usual Care but changes have been made **since the last assessment**
If 'Yes', proceed to Question 4

☐ Patient has changed from NPWT to Usual Care **since the last assessment**
If 'Yes', proceed to Question 4

☐ Patient has changed from Usual Care to NPWT **since the last assessment**
If 'Yes', proceed to Question 4

4. Please indicate why the patient has had changes made since the last assessment *(cross all that apply)*:

☐ Wound improving ☐ Wound healed ☐ Wound bed prepared

☐ Wound too dry ☐ Deterioration of wound ☐ Failure to maintain seal

☐ Treatment caused pain ☐ No change to wound

☐ Other (please specify): ____________________

Was the change at: ☐ Patient request ☐ Clinician request

If participant is receiving NPWT, proceed to Section C

If participant is receiving Usual Care, proceed to Section D

Participant ID: ☐☐☐☐

Section C – Changes to NPWT Treatment Please only complete if you have selected 3c or 3f

Please provide date change made: ☐☐ / ☐☐ / ☐☐☐☐ (day / month / year)

Please provide details of the NPWT treatment the patient is now receiving *(select one option)*:

1. ☐ V.A.C Ulta (KCI) ☐ V.A.C Via (KCI)
   ☐ InfoV.A.C (KCI) ☐ ActiV.A.C (KCI)
   ☐ Renasys (Smith and Nephew) ☐ PICO (Smith and Nephew)
   ☐ Avance (Molnlycke Health Care) ☐ Avance Solo (Molnlycke Health Care)
   ☐ Avelle (Convatec)
   ☐ Other (please specify model and brand): ____________

2. Amount of pressure being used: ☐☐☐ *mmHg*

3. Is the pressure being applied: ☐ Continuously ☐ Intermittently

4. Type of NPWT dressing being used:
   ☐ Black, polyurethane foam dressings with reticulated (open) pores
   ☐ White, polyvinyl alcohol with high tensile strength, pre-moistened with sterile water
   ☐ Antimicrobial gauze (impregnated with Polyhexamethylene biguanide)

5. Has a liner been used with the dressing? ☐ Yes ☐ No

a) If 'Yes', was the liner silver impregnated? ☐ Yes ☐ No

Please proceed to Section E

Section D – Changes to Usual Care Treatment Please only complete if you have selected 3d or 3e

Please provide date change made: ☐☐ / ☐☐ / ☐☐☐☐ (day / month / year)

Please provide details of the Usual Care treatment the patient is now receiving *(cross all that apply)*:

☐ Alginate ☐ Basic wound contact
☐ Cavity Foam ☐ Foam
☐ Gauze Ribbon ☐ Hydrocolloid
☐ Hydrogel ☐ Hydrofoam/Spun Hydrocolloid
☐ Honey ☐ Iodine Containing
☐ PHMB ☐ Protease Modulating matrix
☐ Silver containing ☐ Flamazine silver sulfadiazine dressing
☐ Urgotol silver sulfadiazine dressing ☐ Soft polymer
☐ Superabsorbent ☐ Vapour permeable film or membrane
☐ Other (please specify): ____________
☐ Other (please specify): ____________

Please proceed to Section E

Section E – Other Treatments

1. Is the participant currently using antibiotics? ☐ Yes ☐ No

a) If ‘Yes’, please indicate the route of administration:

☐ IV ☐ Oral ☐ Other (please specify): ______

b) If ‘Yes’, please indicate the reason:

☐ Related to SWHSI ☐ Other (please specify): ______

2. Is the participant receiving any other treatment for their SWHSI? ☐ Yes ☐ No

a) If 'Yes', please provide details below *(cross all that apply)*:

☐ Wound Management Systems ☐ Emollient ☐ Silver Nitrate Stick

☐ Dietetic Input ☐ Larvae

☐ Other (please specify): ______

☐ Other (please specify): ______

☐ Other (please specify): ______

Please proceed to Section F

Section F – Visit Checklist and Sign Off

1. All Sections of the Weekly Assessment are complete? ☐ Yes ☐ No

2. Has the next weekly assessment (or first post wound healing visit if wound has healed) been arranged? ☐ Yes ☐ No

Form Completed by (to be completed by delegated clinician or nurse who has reviewed CRF content for this visit)

*Name: ______

Signature: ______

Assessor ID: ☐☐

Date: ☐☐ / ☐☐ / ☐☐☐☐
day month year

*Must be reflected in the Delegation of Duties Log

Participant ID: ☐☐☐☐ **Visit Date:** ☐☐ / ☐☐ / ☐☐☐☐ (day / month / year)

Section A – Clinical Events

Please review any previous assessments and AE/SAE forms for ongoing events before completing this section.

Have any of the following events occurred since the last assessment?

Section A1 – Wound Healing

Event	Yes	No	Event Date *(dd/mm/yyyy)*
1) Has healing of the reference SWHSI (confirmed by a healthcare professional) occurred since the last visit?	☐	☐	☐☐ / ☐☐ / ☐☐☐☐
a) If 'No', does the participant think their wound has healed?	☐	☐	

Section A2 – Clinical Events

Event	Yes	No	Event Date *(dd/mm/yyyy)*
1) Did the participant have an infected SWHSI at the last assessment?	☐	☐	
a) If 'Yes', has the infection resolved?	☐	☐	Date of Infection Resolution: ☐☐ / ☐☐ / ☐☐☐☐
2) Has the participant had a new SWHSI infection since their last assessment?	☐	☐	Date of New Infection: ☐☐ / ☐☐ / ☐☐☐☐
If 'Yes'... a) Has a SWHSI-2 Trial Adverse Event form been completed?	☐	☐	Adverse Event Number ☐☐
b) Has the new infection been resolved?	☐	☐	Date of Infection Resolution: ☐☐ / ☐☐ / ☐☐☐☐
3) Was the patient an inpatient in relation to their SWHSI or SWHSI treatment at the last assessment	☐	☐	
a) If 'Yes', has the participant been discharged?	☐	☐	Date of Discharge: ☐☐ / ☐☐ / ☐☐☐☐
4) Has the participant been admitted as an inpatient in relation to their SWHSI or SWHSI treatment since the last assessment?	☐	☐	Date of Admission: ☐☐ / ☐☐ / ☐☐☐☐
If 'Yes'... a) Has a SWHSI-2 Trial Adverse Event form been completed?	☐	☐	Adverse Event Number ☐☐
b) Has the participant been discharged?	☐	☐	Date of Discharge: ☐☐ / ☐☐ / ☐☐☐☐

5) Return to theatre due to SWHSI ☐ Yes ☐ No ☐☐ / ☐☐ / ☐☐☐☐ (dd / mm / yyyy)

a) If 'Yes', in theatre was the participant's SWHSI *(cross all that apply)*:

☐ Surgically closed (flap) ☐ Surgically closed (skin graft)
☐ Surgically closed (delayed primary healing) ☐ Debrided ☐ Revascularised
☐ Incised and drained ☐ Examined under anaesthetic ☐ Amputated
☐ Angioplasty ☐ Other (please specify) ____________

b) If 'Yes', has a SWHSI-2 Trial Adverse Event form been completed? ☐ Yes ☐ No Adverse Event Number ☐☐

c) Where the participant's SWHSI was closed, was the decision for closure made by a clinician blind to treatment allocation? ☐ Yes ☐ No

d) If 'No', please indicate why this assessment was not blinded: ____________

Please proceed to Section B

Participant ID: ☐☐☐☐

Section B – Treatment Status (Visits 2-4 only)

1. Has the location of the patient's treatment changed **since the last assessment**? ☐ Yes ☐ No

a) If 'Yes', please select where the patient is currently receiving treatment *(cross all that apply)*:

☐ Community Clinic ☐ Nursing Home ☐ Patient's home

☐ General ward ☐ ICU ☐ High Dependency Unit

☐ Other (please specify): ____________________

2. Please indicate the number of dressing changes the participant has received **since the last assessment** *(select one option)*:

☐ 1 ☐ 2 ☐ 3 ☐ 4 ☐ 5 ☐ 6 ☐ 7 ☐ 8 ☐ 9 ☐ 10

☐ 11 ☐ 12 ☐ 13 ☐ 14 ☐ Other (please specify): ☐☐

3. Please indicate the participants current treatment status *(select one option)*:

☐ Patient continues to receive NPWT with no changes **since the last assessment**
If 'Yes', proceed to Section E

☐ Patient continues to receive Usual Care with no changes **since the last assessment**
If 'Yes', proceed to Section E

☐ Patient continues to receive NPWT but changes have been made **since the last assessment**
If 'Yes', proceed to Question 4

☐ Patient continues to receive Usual Care but changes have been made **since the last assessment**
If 'Yes', proceed to Question 4

☐ Patient has changed from NPWT to Usual Care **since the last assessment**
If 'Yes', proceed to Question 4

☐ Patient has changed from Usual Care to NPWT **since the last assessment**
If 'Yes', proceed to Question 4

4. Please indicate why the patient has had changes made since the last assessment *(cross all that apply)*:

☐ Wound improving ☐ Wound healed ☐ Wound bed prepared

☐ Wound too dry ☐ Deterioration of wound ☐ Failure to maintain seal

☐ Treatment caused pain ☐ No change to wound

☐ Other (please specify): ____________________

Was the change at: ☐ Patient request ☐ Clinician request

If participant is receiving NPWT, proceed to Section C

If participant is receiving Usual Care, proceed to Section D

Participant ID: ☐☐☐☐

Section C – Changes to NPWT Treatment Please only complete if you have selected 3c or 3f

Please provide date change made: ☐☐ / ☐☐ / ☐☐☐☐
(day / month / year)

Please provide details of the NPWT treatment the patient is now receiving *(select one option)*:

1. ☐ V.A.C Ulta (KCI) ☐ V.A.C Via (KCI)
   ☐ InfoV.A.C (KCI) ☐ ActiV.A.C (KCI)
   ☐ Renasys (Smith and Nephew) ☐ PICO (Smith and Nephew)
   ☐ Avance (Molnlycke Health Care) ☐ Avance Solo (Molnlycke Health Care)
   ☐ Avelle (Convatec)
   ☐ Other (please specify model and brand): ____________

2. Amount of pressure being used: ☐☐☐ *mmHg*

3. Is the pressure being applied: ☐ Continuously ☐ Intermittently

4. Type of NPWT dressing being used:
   ☐ Black, polyurethane foam dressings with reticulated (open) pores
   ☐ White, polyvinyl alcohol with high tensile strength, pre-moistened with sterile water
   ☐ Antimicrobial gauze (impregnated with Polyhexamethylene biguanide)

5. Has a liner been used with the dressing? ☐ Yes ☐ No

a) If 'Yes', was the liner silver impregnated? ☐ Yes ☐ No

Please proceed to Section E

Section D – Changes to Usual Care Treatment Please only complete if you have selected 3d or 3e

Please provide date change made: ☐☐ / ☐☐ / ☐☐☐☐
(day / month / year)

Please provide details of the Usual Care treatment the patient is now receiving *(cross all that apply)*:

☐ Alginate ☐ Basic wound contact
☐ Cavity Foam ☐ Foam
☐ Gauze Ribbon ☐ Hydrocolloid
☐ Hydrogel ☐ Hydrofoam/Spun Hydrocolloid
☐ Honey ☐ Iodine Containing
☐ PHMB ☐ Protease Modulating matrix
☐ Silver containing ☐ Flamazine silver sulfadiazine dressing
☐ Urgotol silver sulfadiazine dressing ☐ Soft polymer
☐ Superabsorbent ☐ Vapour permeable film or membrane
☐ Other (please specify): ____________
☐ Other (please specify): ____________

Please proceed to Section E

Section E – Other Treatments

1. Is the participant currently using antibiotics? ☐ Yes ☐ No

a) If 'Yes', please indicate the route of administration:

☐ IV ☐ Oral ☐ Other (please specify): ________

b) If 'Yes', please indicate the reason:

☐ Related to SWHSI ☐ Other (please specify): ________

2. Is the participant receiving any other treatment for their SWHSI? ☐ Yes ☐ No

a) If 'Yes', please provide details below *(cross all that apply)*:

☐ Wound Management Systems ☐ Emollient ☐ Silver Nitrate Stick

☐ Dietetic Input ☐ Larvae

☐ Other (please specify): ________

☐ Other (please specify): ________

☐ Other (please specify): ________

Please proceed to Section F

Section F – Visit Checklist and Sign Off

1. All Sections of the Weekly Assessment are complete? ☐ Yes ☐ No

2. Has the next weekly assessment (or first post wound healing visit if wound has healed) been arranged? ☐ Yes ☐ No

Form Completed by (to be completed by delegated clinician or nurse who has reviewed CRF content for this visit)

*Name: ________

Signature: ________

Assessor ID: ☐☐

Date: ☐☐ / ☐☐ / ☐☐☐☐
day month year

*Must be reflected in the Delegation of Duties Log

Participant ID: ☐☐☐☐ **Visit Date:** ☐☐ / ☐☐ / ☐☐☐☐
day *month* *year*

Section A – Clinical Events

Please review any previous assessments and AE/SAE forms for ongoing events before completing this section.

Have any of the following events occurred since the last assessment?

Section A1 – Wound Healing

Event	Yes	No	Event Date *(dd/mm/yyyy)*
1) Has healing of the reference SWHSI (confirmed by a healthcare professional) occurred since the last visit?	☐	☐	☐☐ / ☐☐ / ☐☐☐☐
a) If 'No', does the participant think their wound has healed?	☐	☐	

Section A2 – Clinical Events

Event	Yes	No	Event Date *(dd/mm/yyyy)*
1) Did the participant have an infected SWHSI at the last assessment?	☐	☐	
a) If 'Yes', has the infection resolved?	☐	☐	Date of Infection Resolution: ☐☐ / ☐☐ / ☐☐☐☐
2) Has the participant had a new SWHSI infection since their last assessment?	☐	☐	Date of New Infection: ☐☐ / ☐☐ / ☐☐☐☐
If 'Yes'... a) Has a SWHSI-2 Trial Adverse Event form been completed?	☐	☐	Adverse Event Number ☐☐
b) Has the new infection been resolved?	☐	☐	Date of Infection Resolution: ☐☐ / ☐☐ / ☐☐☐☐
3) Was the patient an inpatient in relation to their SWHSI or SWHSI treatment at the last assessment	☐	☐	
a) If 'Yes', has the participant been discharged?	☐	☐	Date of Discharge: ☐☐ / ☐☐ / ☐☐☐☐
4) Has the participant been admitted as an inpatient in relation to their SWHSI or SWHSI treatment since the last assessment?	☐	☐	Date of Admission: ☐☐ / ☐☐ / ☐☐☐☐
If 'Yes'... a) Has a SWHSI-2 Trial Adverse Event form been completed?	☐	☐	Adverse Event Number ☐☐
b) Has the participant been discharged?	☐	☐	Date of Discharge: ☐☐ / ☐☐ / ☐☐☐☐

5) Return to theatre due to SWHSI ☐ Yes ☐ No ☐☐ / ☐☐ / ☐☐☐☐ (*dd* *mm* *yyyy*)

a) If 'Yes', in theatre was the participant's SWHSI *(cross all that apply)*:

- ☐ Surgically closed (flap)
- ☐ Surgically closed (skin graft)
- ☐ Surgically closed (delayed primary healing)
- ☐ Debrided
- ☐ Revascularised
- ☐ Incised and drained
- ☐ Examined under anaesthetic
- ☐ Amputated
- ☐ Angioplasty
- ☐ Other (please specify) ________

b) If 'Yes', has a SWHSI-2 Trial Adverse Event form been completed? ☐ Yes ☐ No Adverse Event Number ☐☐

c) Where the participant's SWHSI was closed, was the decision for closure made by a clinician blind to treatment allocation? ☐ Yes ☐ No

d) If 'No', please indicate why this assessment was not blinded: ________

Please proceed to Section B

Section B – Treatment Status (Visits 2-4 only)

1. Has the location of the patient's treatment changed s**ince the last assessment**? ☐ Yes ☐ No

a) If 'Yes', please select where the patient is currently receiving treatment *(cross all that apply)*:

☐ Community Clinic ☐ Nursing Home ☐ Patient's home

☐ General ward ☐ ICU ☐ High Dependency Unit

☐ Other (please specify): ____________________

2. Please indicate the number of dressing changes the participant has received **since the last assessment** *(select one option)*:

☐ 1 ☐ 2 ☐ 3 ☐ 4 ☐ 5 ☐ 6 ☐ 7 ☐ 8 ☐ 9 ☐ 10

☐ 11 ☐ 12 ☐ 13 ☐ 14 ☐ Other (please specify): ☐☐

3. Please indicate the participants current treatment status *(select one option)*:

☐ Patient continues to receive NPWT with no changes **since the last assessment**
If 'Yes', proceed to Section E

☐ Patient continues to receive Usual Care with no changes **since the last assessment**
If 'Yes', proceed to Section E

☐ Patient continues to receive NPWT but changes have been made **since the last assessment**
If 'Yes', proceed to Question 4

☐ Patient continues to receive Usual Care but changes have been made **since the last assessment**
If 'Yes', proceed to Question 4

☐ Patient has changed from NPWT to Usual Care **since the last assessment**
If 'Yes', proceed to Question 4

☐ Patient has changed from Usual Care to NPWT **since the last assessment**
If 'Yes', proceed to Question 4

4. Please indicate why the patient has had changes made since the last assessment *(cross all that apply)*:

☐ Wound improving ☐ Wound healed ☐ Wound bed prepared

☐ Wound too dry ☐ Deterioration of wound ☐ Failure to maintain seal

☐ Treatment caused pain ☐ No change to wound

☐ Other (please specify): ____________________

Was the change at: ☐ Patient request ☐ Clinician request

If participant is receiving NPWT, proceed to Section C

If participant is receiving Usual Care, proceed to Section D

Participant ID: ☐☐☐☐

Section C – Changes to NPWT Treatment Please only complete if you have selected 3c or 3f

Please provide date change made: ☐☐ / ☐☐ / ☐☐☐☐
day month year

Please provide details of the NPWT treatment the patient is now receiving *(select one option)*:

1. ☐ V.A.C Ulta (KCI) ☐ V.A.C Via (KCI)
   ☐ InfoV.A.C (KCI) ☐ ActiV.A.C (KCI)
   ☐ Renasys (Smith and Nephew) ☐ PICO (Smith and Nephew)
   ☐ Avance (Molnlycke Health Care) ☐ Avance Solo (Molnlycke Health Care)
   ☐ Avelle (Convatec)
   ☐ Other (please specify model and brand): ____________

2. Amount of pressure being used: ☐☐☐ *mmHg*
3. Is the pressure being applied: ☐ Continuously ☐ Intermittently
4. Type of NPWT dressing being used:
   ☐ Black, polyurethane foam dressings with reticulated (open) pores
   ☐ White, polyvinyl alcohol with high tensile strength, pre-moistened with sterile water
   ☐ Antimicrobial gauze (impregnated with Polyhexamethylene biguanide)
5. Has a liner been used with the dressing? ☐ Yes ☐ No

a) If 'Yes', was the liner silver impregnated? ☐ Yes ☐ No

Please proceed to Section E

Section D – Changes to Usual Care Treatment Please only complete if you have selected 3d or 3e

Please provide date change made: ☐☐ / ☐☐ / ☐☐☐☐
day month year

Please provide details of the Usual Care treatment the patient is now receiving *(cross all that apply)*:

☐ Alginate ☐ Basic wound contact
☐ Cavity Foam ☐ Foam
☐ Gauze Ribbon ☐ Hydrocolloid
☐ Hydrogel ☐ Hydrofoam/Spun Hydrocolloid
☐ Honey ☐ Iodine Containing
☐ PHMB ☐ Protease Modulating matrix
☐ Silver containing ☐ Flamazine silver sulfadiazine dressing
☐ Urgotol silver sulfadiazine dressing ☐ Soft polymer
☐ Superabsorbent ☐ Vapour permeable film or membrane
☐ Other (please specify): ____________
☐ Other (please specify): ____________

Please proceed to Section E

Participant ID: ☐☐☐☐

Section E – Other Treatments

1. Is the participant currently using antibiotics? ☐ Yes ☐ No

a) If 'Yes', please indicate the route of administration:

☐ IV ☐ Oral ☐ Other (please specify): ____________________

b) If 'Yes', please indicate the reason:

☐ Related to SWHSI ☐ Other (please specify): ____________________

2. Is the participant receiving any other treatment for their SWHSI? ☐ Yes ☐ No

a) If 'Yes', please provide details below *(cross all that apply)*:

☐ Wound Management Systems ☐ Emollient ☐ Silver Nitrate Stick

☐ Dietetic Input ☐ Larvae

☐ Other (please specify): ____________________

☐ Other (please specify): ____________________

☐ Other (please specify): ____________________

Please proceed to Section F

Section F – Visit Checklist and Sign Off

1. All Sections of the Weekly Assessment are complete? ☐ Yes ☐ No

2. Has the next weekly assessment (or first post wound healing visit if wound has healed) been arranged? ☐ Yes ☐ No

Form Completed by (to be completed by delegated clinician or nurse who has reviewed CRF content for this visit)

*Name: ____________________

Signature: ____________________

Assessor ID: ☐☐

Date: ☐☐ / ☐☐ / ☐☐☐☐
day *month* *year*

*Must be reflected in the Delegation of Duties Log
