# Supplementary material for: Negative pressure wound therapy versus usual care for surgical wounds healing by secondary intention (SWHSI-2 trial): study protocol for a pragmatic, multicentre, cross surgical specialty, randomised controlled trial
Source: Trials. 2021 Oct 25;22:739. doi: 10.1186/s13063-021-05662-2 (PMC8543414; doi:10.1186/s13063-021-05662-2)
Supplement: Supplementary file 4 — Additional file 4. Treatment Delivery Case Report Form [file 13063_2021_5662_MOESM4_ESM.pdf]

UNIVERSITY of York
The Department of Health Sciences

NHS
Hull University Teaching Hospitals
NHS Trust

SWHSI-2 Trial

Treatment Delivery Case Report Form

FOR STUDY INVESTIGATOR COMPLETION

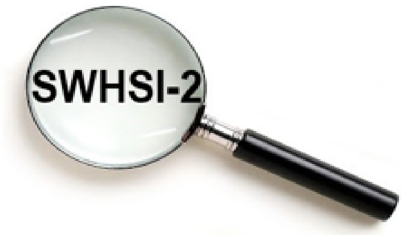

Site ID: ☐☐

Participant ID: ☐☐☐☐

Assessment Date: ☐☐ / ☐☐ / ☐☐☐☐
day *month* *year*

Instructions for Completion

This CRF may be completed by the principal investigator or a delegated member of staff listed on the SWHSI-2 Trial Delegation Log.

Please refer to the associated SWHSI-2 Trial Specific Procedure for full details of how to complete this CRF.

Please complete all sections of this questionnaire using the spaces provided, and sign off when complete.

Please do not include any patient identifiable information when completing this CRF. When complete, please remove the staple and take a photocopy of the completed CRF for your site records. **Please do not re-staple the original.** Place the unstapled original in a "SWHSI-2 Trial business reply envelope" and send via post to York Trials Unit.

This project is funded by the National Institute for Health Research Health Technology Assessment Programme (Project number 17/42/94)

Section A – Pre Treatment Confirmation

1. Please confirm the treatment the participant was randomised to receive:

☐ Intervention (NPWT) ☐ Usual Care (Standard Care)

2. Is the assigned treatment going to be applied? ☐ Yes ☐ No

If 'Yes', and allocated to Usual Care, please proceed to Section B. If allocated to NPWT please proceed to Section C.

If 'No', please provide a reason, complete a change of status form (if required) and provide details of treatment received in the relevant section below.

Section B – Usual Care Details

1. Please select treatment being used following randomisation: *(select all applicable)*

☐ Alginate
☐ Basic wound contact
☐ Cavity Foam
☐ Foam
☐ Gauze Ribbon
☐ Hydrocolloid
☐ Hydrogel
☐ Hydrofoam/Spun Hydrocolloid
☐ Honey
☐ Iodine Containing
☐ PHMB
☐ Protease Modulating matrix
☐ Silver containing
☐ Flamazine silver sulfadiazine dressing
☐ Urgotol silver sulfadiazine dressing
☐ Soft polymer
☐ Superabsorbent
☐ Vapour permeable film or membrane

☐ Other (please specify): ____________

☐ Other (please specify): ____________

Please proceed to Section C

Participant ID: ☐☐☐☐

Section C – NPWT Details

1. Date NPWT treatment started: ☐☐ / ☐☐ / ☐☐☐☐ (day / month / year)

2. Was treatment provided within 48 hours of randomisation? ☐ Yes ☐ No

   If 'Yes', please proceed to Question 3

   If 'No', please provide reason for treatment delay:

   - ☐ NPWT machine unavailable
   - ☐ NPWT pump on order
   - ☐ Trained staff member unavailable
   - ☐ Patient unwilling to wait for treatment prior to discharge
   - ☐ Patient moving to different care provider (e.g. from hospital to community care)
   - ☐ Other (please specify): ____________

3. Type of machine used:

   - ☐ V.A.C Ulta (KCI)
   - ☐ V.A.C Via (KCI)
   - ☐ InfoV.A.C (KCI)
   - ☐ ActiV.A.C (KCI)
   - ☐ Renasys (Smith and Nephew)
   - ☐ PICO (Smith and Nephew)
   - ☐ Avance (Molnlycke Health Care)
   - ☐ Avance Solo (Molnlycke Health Care)
   - ☐ Avelle (Convatec)
   - ☐ Other (please specify model and brand): ____________

4. Amount of pressure being used: ☐☐☐ *mmHg*

5. Is the pressure being applied: ☐ Continuously ☐ Intermittently

6. Type of NPWT dressing being used:

   - ☐ Black, polyurethane foam dressings with reticulated (open) pores
   - ☐ White, polyvinyl alcohol with high tensile strength, pre-moistened with sterile water
   - ☐ Antimicrobial gauze (impregnated with Polyhexamethylene biguanide)

7. Has a liner been used with the dressing? ☐ Yes ☐ No

   If 'Yes', was the liner silver impregnated? ☐ Yes ☐ No

Please proceed to Section D

Participant ID: ☐☐☐☐

Section D – Other Treatments

1. Is the participant receiving any other treatment for their SWHSI? ☐ Yes ☐ No

If 'Yes', please provide details below:

☐ Wound Management Systems ☐ Emollient ☐ Silver Nitrate Stick

☐ Dietetic Input ☐ Larvae

☐ Other (please specify): ____________________

☐ Other (please specify): ____________________

☐ Other (please specify): ____________________

Please proceed to Section E

Section E – Checklist and CRF Sign Off

All Sections of the Treatment Delivery CRF are complete ☐ Yes ☐ No

Form Completed by: (to be completed by delegated clinician or nurse who has reviewed CRF content)

*Name: ____________________

Signature: ____________________

Assessor ID: ☐☐

Date: ☐☐ / ☐☐ / ☐☐☐☐
day *month* *year*

*Must be reflected in the Delegation of Duties Log
