# Supplementary material for: Negative pressure wound therapy versus usual care for surgical wounds healing by secondary intention (SWHSI-2 trial): study protocol for a pragmatic, multicentre, cross surgical specialty, randomised controlled trial
Source: Trials. 2021 Oct 25;22:739. doi: 10.1186/s13063-021-05662-2 (PMC8543414; doi:10.1186/s13063-021-05662-2)
Supplement: Supplementary file 5 — Additional file 5. SWHSI-2 Supplementary Page for Outpatient Visits [file 13063_2021_5662_MOESM5_ESM.pdf]

Participant ID: ☐☐☐☐

SWHSI-2 Supplementary Page for Outpatient Visits

Please continue to record any outpatient visits (in addition to those recorded in the 12M Resource Use CRF)

Visit Date	Clinic Type	Other Treatment Provided	Adverse Event
☐☐ / ☐☐ / ☐☐☐☐<br>Day Month Year	Diabetic Foot Clinic ☐ Occupational Therapy ☐<br>Pain Management ☐ Physiotherapy ☐<br>Podiatry ☐ Post Surgical Review ☐<br>Speciality Dressing Clinic ☐ Tissue Viability ☐<br>Other, please specify ☐	Yes ☐ No ☐<br>If Yes, please stick all that apply:<br>Larvae ☐ Analgesia ☐ Antibiotic ☐<br>Dietetic Input ☐ Topical Treatments ☐<br>Wound Management Systems ☐<br>Other, please specify ☐	Reported?<br>Yes ☐ No ☐<br>AE Number<br>☐☐
☐☐ / ☐☐ / ☐☐☐☐<br>Day Month Year	Diabetic Foot Clinic ☐ Occupational Therapy ☐<br>Pain Management ☐ Physiotherapy ☐<br>Podiatry ☐ Post Surgical Review ☐<br>Speciality Dressing Clinic ☐ Tissue Viability ☐<br>Other, please specify ☐	Yes ☐ No ☐<br>If Yes, please stick all that apply:<br>Larvae ☐ Analgesia ☐ Antibiotic ☐<br>Dietetic Input ☐ Topical Treatments ☐<br>Wound Management Systems ☐<br>Other, please specify ☐	Reported?<br>Yes ☐ No ☐<br>AE Number<br>☐☐

Visit Date	Clinic Type	Other Treatment Provided	Adverse Event
☐☐ / ☐☐ / ☐☐☐☐<br>*Day* *Month* *Year*	Diabetic Foot Clinic ☐ Occupational Therapy ☐<br>Pain Management ☐ Physiotherapy ☐<br>Podiatry ☐ Post Surgical Review ☐<br>Speciality Dressing Clinic ☐ Tissue Viability ☐<br>Other, please specify ☐	Yes ☐ No ☐<br>If Yes, please stick all that apply:<br>Larvae ☐ Analgesia ☐ Antibiotic ☐<br>Dietetic Input ☐ Topical Treatments ☐<br>Wound Management Systems ☐<br>Other, please specify ☐	Reported?<br>Yes ☐ No ☐<br>AE Number<br>☐☐
☐☐ / ☐☐ / ☐☐☐☐<br>*Day* *Month* *Year*	Diabetic Foot Clinic ☐ Occupational Therapy ☐<br>Pain Management ☐ Physiotherapy ☐<br>Podiatry ☐ Post Surgical Review ☐<br>Speciality Dressing Clinic ☐ Tissue Viability ☐<br>Other, please specify ☐	Yes ☐ No ☐<br>If Yes, please stick all that apply:<br>Larvae ☐ Analgesia ☐ Antibiotic ☐<br>Dietetic Input ☐ Topical Treatments ☐<br>Wound Management Systems ☐<br>Other, please specify ☐	Reported?<br>Yes ☐ No ☐<br>AE Number<br>☐☐

Form Completed by:

Name: ______________________________ Assessor ID: ☐☐ Date: ☐☐ / ☐☐ / 2 0 ☐☐

Day *Month* *Year*
