# Supplementary material for: Negative pressure wound therapy versus usual care for surgical wounds healing by secondary intention (SWHSI-2 trial): study protocol for a pragmatic, multicentre, cross surgical specialty, randomised controlled trial
Source: Trials. 2021 Oct 25;22:739. doi: 10.1186/s13063-021-05662-2 (PMC8543414; doi:10.1186/s13063-021-05662-2)
Supplement: Supplementary file 6 — Additional file 6. SWHSI-2 Supplementary Page for Inpatient Admissions [file 13063_2021_5662_MOESM6_ESM.pdf]

Participant ID: ☐☐☐☐

SWHSI-2 Supplementary Page for Inpatient Admissions

Please continue to record any inpatient admissions (in addition to those recorded in the 12M Resource Use CRF)

Admission Date	Surgical Intervention Provided	Other Treatment Provided	Discharge Date	Adverse Event
☐☐ / ☐☐ / ☐☐☐☐<br>*Day* *Month* *Year*	Yes ☐ No ☐<br>If Yes, please tick all that apply:<br>Amputation ☐ Angloplasty ☐<br>Incision & Drainage ☐ Debridement ☐<br>Revascularisation ☐ Wound Closure ☐<br>☐ Other, please specify	Yes ☐ No ☐<br>If Yes, please tick all that apply:<br>Analgesia ☐ Antibiotic ☐<br>Larvae ☐ Topical Treatments ☐<br>Wound Management Systems ☐<br>☐ Other, please specify	☐☐ / ☐☐ / ☐☐☐☐<br>*Day* *Month* *Year*	Reported?<br>Yes ☐ No ☐<br>AE Number<br>☐☐
☐☐ / ☐☐ / ☐☐☐☐<br>*Day* *Month* *Year*	Yes ☐ No ☐<br>If Yes, please tick all that apply:<br>Amputation ☐ Angloplasty ☐<br>Incision & Drainage ☐ Debridement ☐<br>Revascularisation ☐ Wound Closure ☐<br>☐ Other, please specify	Yes ☐ No ☐<br>If Yes, please tick all that apply:<br>Analgesia ☐ Antibiotic ☐<br>Larvae ☐ Topical Treatments ☐<br>Wound Management Systems ☐<br>☐ Other, please specify	☐☐ / ☐☐ / ☐☐☐☐<br>*Day* *Month* *Year*	Reported?<br>Yes ☐ No ☐<br>AE Number<br>☐☐

Admission Date	Surgical Intervention Provided	Other Treatment Provided	Discharge Date	Adverse Event
☐☐ / ☐☐ / ☐☐☐☐<br>*Day* *Month* *Year*	Yes ☐ No ☐<br>If Yes, please tick all that apply:<br>Amputation ☐ Angloplasty ☐<br>Incision & Drainage ☐ Debridement ☐<br>Revascularisation ☐ Wound Closure ☐<br>☐ Other, please specify	Yes ☐ No ☐<br>If Yes, please tick all that apply:<br>Analgesia ☐ Antibiotic ☐<br>Larvae ☐ Topical Treatments ☐<br>Wound Management Systems ☐<br>☐ Other, please specify	☐☐ / ☐☐ / ☐☐☐☐<br>*Day* *Month* *Year*	Reported?<br>Yes ☐ No ☐<br>AE Number<br>☐☐
☐☐ / ☐☐ / ☐☐☐☐<br>*Day* *Month* *Year*	Yes ☐ No ☐<br>If Yes, please tick all that apply:<br>Amputation ☐ Angloplasty ☐<br>Incision & Drainage ☐ Debridement ☐<br>Revascularisation ☐ Wound Closure ☐<br>☐ Other, please specify	Yes ☐ No ☐<br>If Yes, please tick all that apply:<br>Analgesia ☐ Antibiotic ☐<br>Larvae ☐ Topical Treatments ☐<br>Wound Management Systems ☐<br>☐ Other, please specify	☐☐ / ☐☐ / ☐☐☐☐<br>*Day* *Month* *Year*	Reported?<br>Yes ☐ No ☐<br>AE Number<br>☐☐

Form Completed by:

Name: ______________________________ Assessor ID: ☐☐ Date: ☐☐ / ☐☐ / 2 0 ☐☐
Day *Month* *Year*
