# Supplementary material for: Negative pressure wound therapy versus usual care for surgical wounds healing by secondary intention (SWHSI-2 trial): study protocol for a pragmatic, multicentre, cross surgical specialty, randomised controlled trial
Source: Trials. 2021 Oct 25;22:739. doi: 10.1186/s13063-021-05662-2 (PMC8543414; doi:10.1186/s13063-021-05662-2)
Supplement: Supplementary file 7 — Additional file 7. SWHSI-2 Supplementary Page for Accident and Emergency Visits [file 13063_2021_5662_MOESM7_ESM.pdf]

Participant ID: ☐☐☐☐

# SWHSI-2 Supplementary Page for Accident and Emergency Visits

Please continue to record any Accident and Emergency visits (in addition to those recorded in the 12M Resource Use CRF)

| Visit Date | Intervention provided | Was the patient admitted as an inpatient following the visit? | Adverse Event |
|---|---|---|---|
| ☐☐ / ☐☐ / ☐☐☐☐<br>*Day* *Month* *Year* | ☐ Antibiotic ☐ Analgesia ☐ Larvae<br>☐ Topical Treatments<br>☐ Wound Management Systems<br>☐ Other, please specify | Yes ☐ No ☐ | Reported?<br>Yes ☐ No ☐<br>AE Number<br>☐☐ |
| ☐☐ / ☐☐ / ☐☐☐☐<br>*Day* *Month* *Year* | ☐ Antibiotic ☐ Analgesia ☐ Larvae<br>☐ Topical Treatments<br>☐ Wound Management Systems<br>☐ Other, please specify | Yes ☐ No ☐ | Reported?<br>Yes ☐ No ☐<br>AE Number<br>☐☐ |
| ☐☐ / ☐☐ / ☐☐☐☐<br>*Day* *Month* *Year* | ☐ Antibiotic ☐ Analgesia ☐ Larvae<br>☐ Topical Treatments<br>☐ Wound Management Systems<br>☐ Other, please specify | Yes ☐ No ☐ | Reported?<br>Yes ☐ No ☐<br>AE Number<br>☐☐ |

| Visit Date | Intervention provided | Was the patient admitted as an inpatient following the visit? | Adverse Event |
|---|---|---|---|
| ☐☐ / ☐☐ / ☐☐☐☐<br>*Day* *Month* *Year* | ☐ Antibiotic ☐ Analgesia ☐ Larvae<br>☐ Topical Treatments<br>☐ Wound Management Systems<br>☐ Other, please specify | Yes ☐ No ☐ | Reported?<br>Yes ☐ No ☐<br>AE Number<br>☐☐ |
| ☐☐ / ☐☐ / ☐☐☐☐<br>*Day* *Month* *Year* | ☐ Antibiotic ☐ Analgesia ☐ Larvae<br>☐ Topical Treatments<br>☐ Wound Management Systems<br>☐ Other, please specify | Yes ☐ No ☐ | Reported?<br>Yes ☐ No ☐<br>AE Number<br>☐☐ |
| ☐☐ / ☐☐ / ☐☐☐☐<br>*Day* *Month* *Year* | ☐ Antibiotic ☐ Analgesia ☐ Larvae<br>☐ Topical Treatments<br>☐ Wound Management Systems<br>☐ Other, please specify | Yes ☐ No ☐ | Reported?<br>Yes ☐ No ☐<br>AE Number<br>☐☐ |

Form Completed by:

Name: ______________________________ Assessor ID: ☐☐ Date: ☐☐ / ☐☐ / 2 0 ☐☐
*Day* *Month* *Year*
