# Supplementary material for: Negative pressure wound therapy versus usual care for surgical wounds healing by secondary intention (SWHSI-2 trial): study protocol for a pragmatic, multicentre, cross surgical specialty, randomised controlled trial
Source: Trials. 2021 Oct 25;22:739. doi: 10.1186/s13063-021-05662-2 (PMC8543414; doi:10.1186/s13063-021-05662-2)
Supplement: Supplementary file 8 — Additional file 8. SWHSI-2 Supplementary Page for Infection CDC Assessment [file 13063_2021_5662_MOESM8_ESM.pdf]

SWHSI-2 Supplementary Page for Infection CDC Assessment

Please continue to record any infection episodes (in addition to those recorded in the Post Healing Assessment CRF).

THIS SECTION MUST NOT BE COMPLETED FOR INFECTIONS PREVIOUSLY REPORTED IN THE POST HEALING ASSESSMENT CRF.

Site ID: ☐☐ **Participant ID:** ☐☐☐☐

Infection Start Date: ☐☐ / ☐☐ / ☐☐☐☐ (dd / mm / yyyy) ☐ Exact ☐ Approx

- ☐ **Abscess or other evidence of infection detected on gross anatomical or hisopathologic exam or imaging test**
- ☐ **Aspirated fluid/swab of surgical site yields organisms and pus cells are present**
- ☐ **Clinician's diagnosis**
- ☐ **Fever (temperature 38°C or more)**
- ☐ **Heat**
- ☐ **Incision spontaneously dehisced or was opened by healthcare professional**
- ☐ **Localised pain and tenderness**
- ☐ **Localised swelling**
- ☐ **Purulent drainage**
- ☐ **Redness**

Infection Start Date: ☐☐ / ☐☐ / ☐☐☐☐ (dd / mm / yyyy) ☐ Exact ☐ Approx

- ☐ **Abscess or other evidence of infection detected on gross anatomical or hisopathologic exam or imaging test**
- ☐ **Aspirated fluid/swab of surgical site yields organisms and pus cells are present**
- ☐ **Clinician's diagnosis**
- ☐ **Fever (temperature 38°C or more)**
- ☐ **Heat**
- ☐ **Incision spontaneously dehisced or was opened by healthcare professional**
- ☐ **Localised pain and tenderness**
- ☐ **Localised swelling**
- ☐ **Purulent drainage**
- ☐ **Redness**

Infection Start Date: ☐☐ / ☐☐ / ☐☐☐☐ (dd / mm / yyyy) ☐ Exact ☐ Approx

- ☐ **Abscess or other evidence of infection detected on gross anatomical or hisopathologic exam or imaging test**
- ☐ **Aspirated fluid/swab of surgical site yields organisms and pus cells are present**
- ☐ **Clinician's diagnosis**
- ☐ **Fever (temperature 38°C or more)**
- ☐ **Heat**
- ☐ **Incision spontaneously dehisced or was opened by healthcare professional**
- ☐ **Localised pain and tenderness**
- ☐ **Localised swelling**
- ☐ **Purulent drainage**
- ☐ **Redness**

Infection Start Date: ☐☐ / ☐☐ / ☐☐☐☐ ☐ Exact ☐ Approx
dd *mm* *yyyy*

- ☐ **Abscess or other evidence of infection detected on gross anatomical or hisopathologic exam or imaging test**
- ☐ **Aspirated fluid/swab of surgical site yields organisms and pus cells are present**
- ☐ **Clinician's diagnosis**
- ☐ **Fever (temperature 38°C or more)**
- ☐ **Heat**
- ☐ **Incision spontaneously dehisced or was opened by healthcare professional**
- ☐ **Localised pain and tenderness**
- ☐ **Localised swelling**
- ☐ **Purulent drainage**
- ☐ **Redness**

Infection Start Date: ☐☐ / ☐☐ / ☐☐☐☐ ☐ Exact ☐ Approx
dd *mm* *yyyy*

- ☐ **Abscess or other evidence of infection detected on gross anatomical or hisopathologic exam or imaging test**
- ☐ **Aspirated fluid/swab of surgical site yields organisms and pus cells are present**
- ☐ **Clinician's diagnosis**
- ☐ **Fever (temperature 38°C or more)**
- ☐ **Heat**
- ☐ **Incision spontaneously dehisced or was opened by healthcare professional**
- ☐ **Localised pain and tenderness**
- ☐ **Localised swelling**
- ☐ **Purulent drainage**
- ☐ **Redness**

Infection Start Date: ☐☐ / ☐☐ / ☐☐☐☐ ☐ Exact ☐ Approx
dd *mm* *yyyy*

- ☐ **Abscess or other evidence of infection detected on gross anatomical or hisopathologic exam or imaging test**
- ☐ **Aspirated fluid/swab of surgical site yields organisms and pus cells are present**
- ☐ **Clinician's diagnosis**
- ☐ **Fever (temperature 38°C or more)**
- ☐ **Heat**
- ☐ **Incision spontaneously dehisced or was opened by healthcare professional**
- ☐ **Localised pain and tenderness**
- ☐ **Localised swelling**
- ☐ **Purulent drainage**
- ☐ **Redness**

Signature of person completing page: ____________________

Date *(dd/mm/yyyy)*: ☐☐ / ☐☐ / ☐☐☐☐

Assessor ID: ☐☐
