# Supplementary material for: Negative pressure wound therapy versus usual care for surgical wounds healing by secondary intention (SWHSI-2 trial): study protocol for a pragmatic, multicentre, cross surgical specialty, randomised controlled trial
Source: Trials. 2021 Oct 25;22:739. doi: 10.1186/s13063-021-05662-2 (PMC8543414; doi:10.1186/s13063-021-05662-2)
Supplement: Supplementary file 9 — Additional file 9. Post Healing Assessment Case Report Form [file 13063_2021_5662_MOESM9_ESM.pdf]

The Department of Health Sciences

Hull University Teaching Hospitals NHS Trust

SWHSI-2 Trial

Post Healing Assessment Case Report Form

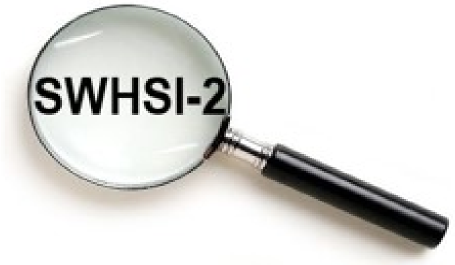

Site ID: ☐☐

Participant ID: ☐☐☐☐

Date of completion: ☐☐ / ☐☐ / ☐☐☐☐
day *month* *year*

Instructions for Completion

This CRF may be completed by the principal investigator or a delegated member of staff listed on the SWHSI-2 Trial Delegation Log. The first section of this Case Report Form (CRF) should be completed by the participant.

Please refer to the associated SWHSI-2 Trial Specific Procedure for full details of how to complete this CRF.

Please complete all sections of this questionnaire using the spaces provided, and sign off when each visit is complete.

Please do not include any patient identifiable information when completing this CRF. When complete, please remove the staple and take a photocopy of the completed CRF for your site records. **Please do not re-staple the original.** Place the unstapled original in a “SWHSI-2 Trial business reply envelope” and send via post to York Trials Unit.

This project is funded by the National Institute for Health Research Health Technology Assessment Programme (Project number 17/42/94)

Participant ID: ☐☐☐☐

Section A – Healing Visit 1   Date: ☐☐ / ☐☐ / ☐☐☐☐
(day / month / year)

Please hand the booklet to the participant and ask them to complete the Wound Healing Questionnaire

1) Wound Healing Questionnaire

Since you have had an open wound…

		Not at all	A little	Quite a bit	A lot
1.	Was there redness spreading away from the wound? (erythema/cellulitis)	☐	☐	☐	☐
2.	Was the area around the wound warmer than the surrounding skin?	☐	☐	☐	☐
3.	Has any part of the wound leaked clear fluid? (serous exudate)	☐	☐	☐	☐
4.	Has any part of the wound leaked blood-stained fluid? (haemoserous exudate)	☐	☐	☐	☐
5.	Has any part of the wound leaked thick and yellow/green fluid? (pus/purulent exudate)	☐	☐	☐	☐
6.	Has the area around the wound become swollen?	☐	☐	☐	☐
7.	Has the wound been smelly?	☐	☐	☐	☐
8.	Has the wound been painful to touch?	☐	☐	☐	☐
9.	Have you had, or felt like you have had, a raised temperature or fever? (fever >38°)	☐	☐	☐	☐

		Yes	No
10.	Have you sought advice because of a problem with your wound, other than at a planned follow-up appointment?	☐	☐
11.	Have you been back into hospital for treatment of a problem with your wound?	☐	☐
12.	Have you been given antibiotics for a problem with your wound?	☐	☐
13.	Has your wound been scraped or cut to remove any unwanted tissue? (debridement of wound)	☐	☐
14.	Has your wound been drained? (drained of pus / abscess)	☐	☐
15.	Have you had an operation under general anaesthetic for treatment of a problem with your wound?	☐	☐

Bluebell Wound Healing Questionnaire © University of Bristol 2017
Modified with permission.

Please return the booklet to the research nurse.

2) Has the participant experienced any difficulties completing the Wound Healing Questionnaire?

☐ Yes ☐ No

If 'Yes', please provide reasons:

☐ Questions were difficult to understand

If 'Yes', please provide the associated question numbers: ________

☐ Participant was unable to recall events

☐ Answer options were insufficient

☐ Some questions did not apply If 'Yes', please provide the associated question numbers: ________

☐ Other, please specify: ________

3) CDC assessment

Has the participant experienced any wound infections during the trial? ☐ Yes ☐ No

If 'Yes', how many infections has the participant experienced? ☐☐

Start date of first infection: ☐☐ / ☐☐ / ☐☐☐☐ (day / month / year) Date is: ☐ Exact ☐ Approximate

Which of the following features were present *(please cross all that applied)*:

☐ Abscess or other evidence of infection detected on gross anatomical or hisopathologic exam or imaging test

☐ Aspirated fluid/swab of surgical site yields organisms and pus cells are present

☐ Clinician's diagnosis

☐ Fever (temperature 38°C or more)

☐ Heat

☐ Incision spontaneously dehisced or was opened by healthcare professional

☐ Localised pain and tenderness

☐ Localised swelling

☐ Purulent drainage

☐ Redness

If the participant has had more than 1 infection, please complete a SWHSI-2 Infection CDC Assessment supplementary page.

4) Healing Status

a) Can healing of the reference SWHSI be confirmed? ☐ Yes ☐ No*

If 'Yes', please provide the date of wound healing, as confirmed by a healthcare professional: ☐☐ / ☐☐ / ☐☐☐☐ (day / month / year)

Please then complete Questions b and c, and arrange healing visit 2.

*If 'No', please complete this visit section and revert to weekly assessments until the wound is healed.

b) Has a photograph of the reference SWHSI been obtained? ☐ Yes ☐ No

If 'No', please provide reasons why a photograph has not been taken: ________

c) Visit Completed by:

Name: ________ Signature: ________

Date: ☐☐ / ☐☐ / ☐☐☐☐ (day / month / year)

Participant ID: [ ][ ][ ][ ]

Section B – Healing Visit 2

Date: [ ][ ] / [ ][ ] / [ ][ ][ ][ ]
day *month* *year*

1) Can healing of the reference SWHSI still be confirmed? ☐ Yes ☐ No*

If 'Yes', please then complete Question 2, and arrange healing visit 3.

*If 'No', please revert to weekly assessments until the wound is healed.

2) Visit Completed by:

Name: ____________________ Signature: ____________________

Date: [ ][ ] / [ ][ ] / [ ][ ][ ][ ]
day *month* *year*

Section C – Healing Visit 3

Date: [ ][ ] / [ ][ ] / [ ][ ][ ][ ]
day *month* *year*

1) Can healing of the reference SWHSI still be confirmed? ☐ Yes ☐ No*

If 'Yes', please then complete Question 2.

*If 'No', please revert to weekly assessments until the wound is healed.

2) Visit Completed by:

Name: ____________________ Signature: ____________________

Date: [ ][ ] / [ ][ ] / [ ][ ][ ][ ]
day *month* *year*
