# Supplementary material for: Negative pressure wound therapy versus usual care for surgical wounds healing by secondary intention (SWHSI-2 trial): study protocol for a pragmatic, multicentre, cross surgical specialty, randomised controlled trial
Source: Trials. 2021 Oct 25;22:739. doi: 10.1186/s13063-021-05662-2 (PMC8543414; doi:10.1186/s13063-021-05662-2)
Supplement: Supplementary file 11 — Additional file 11. Participant 3 Month Questionnaire Booklet [file 13063_2021_5662_MOESM11_ESM.pdf]

The Department of Health Sciences

Hull University Teaching Hospitals NHS Trust

CONFIDENTIAL

Surgical Wounds Healing by Secondary Intention – SWHSI-2

Participant 3 Month Questionnaire Booklet

FOR STUDY PARTICIPANT COMPLETION

For office use only

Site ID: ☐☐

Participant ID: ☐☐☐☐

Date questionnaire sent: ☐☐ / ☐☐ / ☐☐☐☐
day month year

This project is funded by the National Institute for Health Research Health Technology Assessment Programme (Project number 17/42/94)

<u>Instructions for this questionnaire booklet</u>

The purpose of this questionnaire booklet is to find out about your surgical wound and the impact it has on your daily activities. The answers you give in this questionnaire booklet will be treated confidentially.

The questions should be answered by either
- putting a tick in a box
- putting a cross on a line

When you have finished, please check that you have answered all questions, and return the questionnaire in the freepost envelope provided.

Please answer ALL the questionnaire. Although some of the questions may not seem relevant to yourself, your answers do give us valuable information.

If you have further questions or need help with filling in this questionnaire booklet, please ask the SWHSI-2 trial nurse or doctor. Alternatively, please contact a member of the trial team, whose details you will find on your SWHSI-2 Trial Patient Information Leaflet.

You will note that certain questions have been repeated, this is deliberate and essential for the study, and we thank you in advance for your cooperation in filling out every section of this questionnaire booklet.

Please turn overleaf to start filling in this questionnaire booklet.

Participant ID: | | | | |

Please complete the boxes below with the date you are completing this questionnaire booklet: __ __ / __ __ / __ __ __ __ (day / month / year)

Section A – Bluebelle Wound Healing Questionnaire

In the past 3 months…

	Not at all	A little	Quite a bit	A lot
1. Was there redness spreading away from the wound? (erythema/cellulitis)	☐	☐	☐	☐
2. Was the area around the wound warmer than the surrounding skin?	☐	☐	☐	☐
3. Has any part of the wound leaked clear fluid? (serous exudate)	☐	☐	☐	☐
4. Has any part of the wound leaked blood-stained fluid? (haemoserous exudate)	☐	☐	☐	☐
5. Has any part of the wound leaked thick and yellow/green fluid? (pus/purulent exudate)	☐	☐	☐	☐
6. Has the area around the wound become swollen?	☐	☐	☐	☐
7. Has the wound been smelly?	☐	☐	☐	☐
8. Has the wound been painful to touch?	☐	☐	☐	☐
9. Have you had, or felt like you have had, a raised temperature or fever? (fever >38°)	☐	☐	☐	☐

	Yes	No
10. Have you sought advice because of a problem with your wound, other than at a planned follow-up appointment?	☐	☐
11. Have you been back into hospital for treatment of a problem with your wound?	☐	☐
12. Have you been given antibiotics for a problem with your wound?	☐	☐
13. Has your wound been scraped or cut to remove any unwanted tissue? (debridement of wound)	☐	☐
14. Has your wound been drained? (drained of pus / abscess)	☐	☐
15. Have you had an operation under general anaesthetic for treatment of a problem with your wound?	☐	☐

Bluebell Wound Healing Questionnaire © University of Bristol 2017
Modified with permission.

Please proceed to Section B

Participant ID: ☐☐☐☐

Section B – EQ-5D-5L

This section asks about your health in general.
Under each heading, please tick the ONE box that best describes your health **TODAY**.

MOBILITY

I have no problems in walking about ☐
I have slight problems in walking about ☐
I have moderate problems in walking about ☐
I have severe problems in walking about ☐
I am unable to walk about ☐

SELF-CARE

I have no problems washing or dressing myself ☐
I have slight problems washing or dressing myself ☐
I have moderate problems washing or dressing myself ☐
I have severe problems washing or dressing myself ☐
I am unable to wash or dress myself ☐

USUAL ACTIVITIES (e.g. work, study, housework, family or leisure activities)

I have no problems doing my usual activities ☐
I have slight problems doing my usual activities ☐
I have moderate problems doing my usual activities ☐
I have severe problems doing my usual activities ☐
I am unable to do my usual activities ☐

PAIN/DISCOMFORT

I have no pain or discomfort ☐
I have slight pain or discomfort ☐
I have moderate pain or discomfort ☐
I have severe pain or discomfort ☐
I have extreme pain or discomfort ☐

ANXIETY/DEPRESSION

I am not anxious or depressed ☐
I am slightly anxious or depressed ☐
I am moderately anxious or depressed ☐
I am severely anxious or depressed ☐
I am extremely anxious or depressed ☐

© EuroQol Research Foundation. EQ-5D™ is a trade mark of the EuroQol Research Foundation.

Participant ID:

- We would like to know how good or bad your health is TODAY.
- The scale is numbered from 0 to 100.
- 100 means the best health you can imagine.

  0 means the worst health you can imagine.
- Mark an X on the scale to indicate how your health is TODAY.
- Now, please write the number you marked on the scale in the box below.

YOUR HEALTH TODAY =

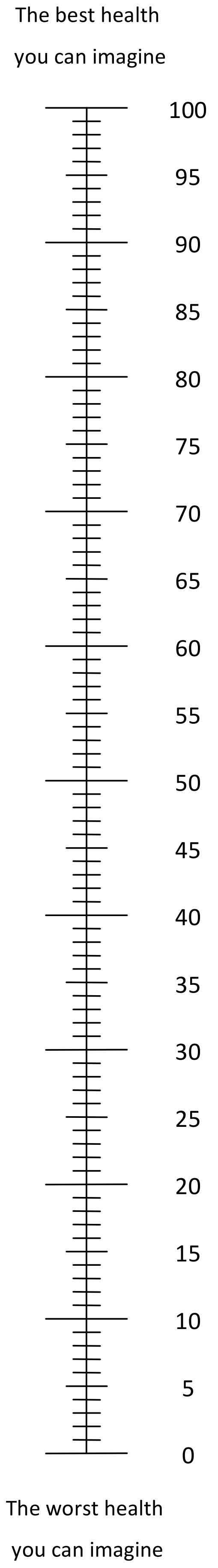

© EuroQol Research Foundation. EQ-5D™ is a trade mark of the EuroQol Research Foundation.

Please proceed to Section C

Participant ID: [ ][ ][ ][ ]

Section C – Pain

We would like to know how good or bad your wound pain is **TODAY**.

The scale below is numbered from 0 to 100. 100 means the worst pain you can imagine and 0 means no pain. Please mark a cross on the line below to indicate how bad your wound pain is today.

No pain |————————————————| **Worst imaginable pain**

0 **100**

[ ][ ][ ]

(for office use only)

Please proceed to Section D

Participant ID: ☐☐☐☐

Section D – Resource Use

This final section asks about the health care you have received <u>related to your wound</u> over the past 3 months.

<u>Care from the NHS NOT in the hospital:</u>

1. **In the past 3 months**, have you had any contact with the following health professionals in the community **<u>IN RELATION TO YOUR WOUND CARE</u>**?

Yes ☐ No ☐

**Have you had contact with**		**Total number of visits (If none enter "0")**
A GP at home	Yes ☐ No ☐	☐☐
A GP at the surgery	Yes ☐ No ☐	☐☐
A nurse at home	Yes ☐ No ☐	☐☐
A nurse at the surgery	Yes ☐ No ☐	☐☐

<u>Care from the NHS IN the hospital:</u>

A) INPATIENT CARE (e.g. admitted and discharged on a different day)

2. **In the past 3 months**, have you been admitted to hospital as an **<u>in-patient</u>** to have further treatment **<u>IN RELATION TO YOUR WOUND</u>**?

Yes ☐ No ☐

Note: Please do not include the visit related to your initial treatment resulting in your wound when you are answering this question.

If 'Yes', how many nights were you in hospital for? ☐☐ nights

B) OUTPATIENT CARE (e.g. not admitted to hospital or discharged on the same day)

3. **In the past 3 months**, have you had any **outpatient visits** to the hospital **IN RELATION TO YOUR WOUND**?

Yes ☐ No ☐

Note: Please do not include the visit related to your initial treatment resulting in your wound when you are answering this question.

If 'Yes', please provide information in the table below:

**Surgical outpatient appointments**	**Had an appointment?**	**Total number of visits**
Vascular, Colorectal or Plastics (e.g. for follow up)	Yes ☐ No ☐	☐☐
Pain Clinic (e.g. for managing pain related to your wound)	Yes ☐ No ☐	☐☐
Physiotherapy (e.g. for rehabilitation)	Yes ☐ No ☐	☐☐
Tissue Viability Service (e.g. for wound review and/or wound dressing)	Yes ☐ No ☐	☐☐
Specialty Dressing Clinic (e.g. for wound review and/or wound dressing)	Yes ☐ No ☐	☐☐
Diabetic Foot Clinic (e.g. for wound review and/or wound dressing)	Yes ☐ No ☐	☐☐
Other visits	Yes ☐ No ☐	
Please provide details of the visit type:		
1) ____________		☐☐
2) ____________		☐☐
3) ____________		☐☐

4. Finally, in the **past 3 months** have you visited **Accident and Emergency (A&E) IN RELATION TO YOUR WOUND**?

Yes ☐ No ☐

If 'Yes', how many times? ☐☐ times

Participant ID: ☐☐☐☐

Return to normal activities

5a. Are you currently employed or self employed?

Yes ☐ No ☐

If 'Yes', are you:

☐ In full time employment (30 hours or more a week)

☐ In part time employment (less than 30 hours a week)

If 'Yes', following your treatment have you been able to return to your paid work?

Yes ☐ No ☐

If 'Yes', approximately how many days after treatment were you able to return to your paid work? ☐☐ days

5b. Following your treatment have you been able to return to any unpaid work you normally do? (e.g. housework, caring duties, voluntary work)

Yes ☐ No ☐

If 'Yes', approximately how many days after treatment were you able to return to your unpaid work? ☐☐ days

5c. Following your treatment have you been able to return to any sporting or social activities which you normally do?

Yes ☐ No ☐

If 'Yes', approximately how many days after treatment were you able to return to your social or sporting activities? ☐☐ days

<u>Private treatments</u>: Could you please tell us about **any additional medical treatments you have received, which you have paid for (e.g. personal cost or personal private insurance health care) <u>IN RELATION TO YOUR WOUND</u>.**

Please also include any private health care paid for by company insurance.

6. Over the **past 3 months**, have you received any private treatments?

Yes ☐ No ☐

If 'Yes', how many times have you:

Seen a doctor for a clinical assessment?<br>*(Please record the number of times in the boxes)*	☐☐<br>*If none enter '0'*
Seen a nurse for a clinical assessment?<br>*(Please record the number of times in the boxes)*	☐☐<br>*If none enter '0'*
Seen a physiotherapist for a clinical assessment or treatment?<br>*(Please record the number of times in the boxes)*	☐☐<br>*If none enter '0'*

<u>Hospital Transport</u>

7. During the **past 3 months**, have you used hospital transport e.g. medicar (or got an allowance for hospital transport) **<u>IN RELATION TO YOUR WOUND</u>**?

Yes ☐ No ☐

If 'Yes', how many times? ☐☐ times Can't remember ☐

Participant ID: ☐☐☐☐

Medications

8. During the **past 3 months**, have you been taking any medications **IN RELATION TO YOUR WOUND**?

Yes ☐ No ☐

If '**Yes**', please cross the box(es) in the table below to indicate the medication that you have taken and whether it has been **prescribed** (by a doctor or other health professional) or you have **bought** the medication yourself. Please also record the **number of days** you have used the medication.

Name of Medication		Number of days	Prescribed	Bought
Paracetamol	☐	☐☐	☐	☐
Co-codamol	☐	☐☐	☐	☐
Tramadol	☐	☐☐	☐	☐
Codeine Phosphate	☐	☐☐	☐	☐
Short acting morphine	☐	☐☐	☐	☐
Long acting morphine	☐	☐☐	☐	☐
Other morphine based tablets	☐	☐☐	☐	☐
Morphine oral suspension	☐	☐☐	☐	☐
Amitriptyline	☐	☐☐	☐	☐
Pregabalin	☐	☐☐	☐	☐
Gabapentin	☐	☐☐	☐	☐
Diclofenac	☐	☐☐	☐	☐
Aspirin	☐	☐☐	☐	☐
Ibuprofen	☐	☐☐	☐	☐
Other, please specify: ______		☐☐	☐	☐
Other, please specify: ______		☐☐	☐	☐

9. Are you a carer or being cared for by others (i.e. family members, care staff)?

☐ Carer

☐ Being cared for by others

☐ Neither of the above

Thank you for taking the time to fill in this questionnaire.
Please check all the pages to make sure that you have answered every statement.

Please return it to the York Trials Unit at the University of York in the pre paid envelope provided.
