# Supplementary material for: Negative pressure wound therapy versus usual care for surgical wounds healing by secondary intention (SWHSI-2 trial): study protocol for a pragmatic, multicentre, cross surgical specialty, randomised controlled trial
Source: Trials. 2021 Oct 25;22:739. doi: 10.1186/s13063-021-05662-2 (PMC8543414; doi:10.1186/s13063-021-05662-2)
Supplement: Supplementary file 12 — Additional file 12. 12 Month Resource Use - Case Report Form [file 13063_2021_5662_MOESM12_ESM.pdf]

SWHSI-2 Trial

12 Month Resource Use - Case Report Form

FOR STUDY INVESTIGATOR COMPLETION

Site ID: ☐☐ **Participant Study Number:** ☐☐☐☐

CRF Completion Date: ☐☐ / ☐☐ / 2 0 ☐☐
Day *Month* *Year*

This project was funded by the National Institute for Health Research Health Technology Assessment Programme (Project number 17/42/94)

This Case Report Form (CRF) may be completed by the Principal Investigator or a delegated member of staff listed on the SWHSI-2 Trial Delegation Log.

Please refer to the associated SWHSI-2 Trial Specific Procedure for full details of how to complete this CRF.

Please complete all sections of this CRF using the spaces provided, and sign off when complete. Please do not include any patient identifiable information when completing this CRF.

When complete, please remove the staple and take a photocopy of the complete CRF for your site records. **Please do not re-staple the original**. Place the unstapled original in a "SWHSI-2 Trial business reply envelope" and send via post to York Trials Unit.

Participant ID: ☐☐☐☐

Section A

Did this participant withdraw from nurse follow up (telephone and/or healing follow up) during the study? ☐ Yes ☐ No

If No, proceed to Section B

If Yes, do patient medical records confirm that the participant's wound healed at any time during the last 12 months? ☐ Yes ☐ No

If Yes, please provide the date of confirmed wound healing: ☐☐ / ☐☐ / ☐☐☐☐ (Day / Month / Year)

If No, does the participant's treating healthcare professional deem the wound to have healed at any time during the last 12 months? ☐ Yes ☐ No

If Yes, please provide the date the healthcare professional confirmed wound healing: ☐☐ / ☐☐ / ☐☐☐☐ (Day / Month / Year)

Proceed to Section B

Section B - Medications

Has the participant been prescribed any medications in the last 12 months for the treatment of their wound? ☐ Yes ☐ No

If Yes, please provide a Yes/No response for **all** of the medications listed below and record the total quantity they were prescribed.

If No, please proceed to Section C.

Drug	Yes	No	Route of Administration	Quantity Unit	Frequency	Total Quantity Prescribed
Analgesia 1	☐	☐	☐ Oral ☐ Subcutaneous ☐ IV ☐ Nasal ☐ IM ☐ Topical ☐ Other (Please specify)	☐ Tablet ☐ Sachet ☐ Vial ☐ Other (Please specify)	☐ Once daily ☐ Twice weekly ☐ Twice daily ☐ PRN ☐ Once weekly ☐ Other (Please specify)	☐☐☐
Analgesia 2	☐	☐	☐ Oral ☐ Subcutaneous ☐ IV ☐ Nasal ☐ IM ☐ Topical ☐ Other (Please specify)	☐ Tablet ☐ Sachet ☐ Vial ☐ Other (Please specify)	☐ Once daily ☐ Twice weekly ☐ Twice daily ☐ PRN ☐ Once weekly ☐ Other (Please specify)	☐☐☐
Analgesia 3	☐	☐	☐ Oral ☐ Subcutaneous ☐ IV ☐ Nasal ☐ IM ☐ Topical ☐ Other (Please specify)	☐ Tablet ☐ Sachet ☐ Vial ☐ Other (Please specify)	☐ Once daily ☐ Twice weekly ☐ Twice daily ☐ PRN ☐ Once weekly ☐ Other (Please specify)	☐☐☐

Participant ID: ☐☐☐☐

Drug	Yes	No	Route of Administration	Quantity Unit	Frequency	Total Quantity Prescribed
Analgesia 4	☐	☐	☐ Oral ☐ Subcutaneous<br>☐ IV ☐ Nasal<br>☐ IM ☐ Topical<br>☐ Other (Please specify)	☐ Tablet<br>☐ Sachet<br>☐ Vial<br>☐ Other (Please specify)	☐ Once daily ☐ Twice weekly<br>☐ Twice daily ☐ PRN<br>☐ Once weekly ☐ Other (Please specify)	☐☐☐
Anticoagulant	☐	☐	☐ Oral ☐ Subcutaneous<br>☐ IV ☐ Nasal<br>☐ IM ☐ Topical<br>☐ Other (Please specify)	☐ Tablet<br>☐ Sachet<br>☐ Vial<br>☐ Other (Please specify)	☐ Once daily ☐ Twice weekly<br>☐ Twice daily ☐ PRN<br>☐ Once weekly ☐ Other (Please specify)	☐☐☐
Antibiotic 1	☐	☐	☐ Oral ☐ Subcutaneous<br>☐ IV ☐ Nasal<br>☐ IM ☐ Topical<br>☐ Other (Please specify)	☐ Tablet<br>☐ Sachet<br>☐ Vial<br>☐ Other (Please specify)	☐ Once daily ☐ Twice weekly<br>☐ Twice daily ☐ PRN<br>☐ Once weekly ☐ Other (Please specify)	☐☐☐
Antibiotic 2	☐	☐	☐ Oral ☐ Subcutaneous<br>☐ IV ☐ Nasal<br>☐ IM ☐ Topical<br>☐ Other (Please specify)	☐ Tablet<br>☐ Sachet<br>☐ Vial<br>☐ Other (Please specify)	☐ Once daily ☐ Twice weekly<br>☐ Twice daily ☐ PRN<br>☐ Once weekly ☐ Other (Please specify)	☐☐☐

Participant ID: ☐☐☐☐

Drug	Yes	No	Route of Administration	Quantity Unit	Frequency	Total Quantity Prescribed
Antibiotic 3	☐	☐	☐ Oral ☐ Subcutaneous<br>☐ IV ☐ Nasal<br>☐ IM ☐ Topical<br>☐ Other (Please specify)	☐ Tablet<br>☐ Sachet<br>☐ Vial<br>☐ Other (Please specify)	☐ Once daily ☐ Twice weekly<br>☐ Twice daily ☐ PRN<br>☐ Once weekly ☐ Other (Please specify)	☐☐☐
Antibiotic 4	☐	☐	☐ Oral ☐ Subcutaneous<br>☐ IV ☐ Nasal<br>☐ IM ☐ Topical<br>☐ Other (Please specify)	☐ Tablet<br>☐ Sachet<br>☐ Vial<br>☐ Other (Please specify)	☐ Once daily ☐ Twice weekly<br>☐ Twice daily ☐ PRN<br>☐ Once weekly ☐ Other (Please specify)	☐☐☐
Antiplatelet	☐	☐	☐ Oral ☐ Subcutaneous<br>☐ IV ☐ Nasal<br>☐ IM ☐ Topical<br>☐ Other (Please specify)	☐ Tablet<br>☐ Sachet<br>☐ Vial<br>☐ Other (Please specify)	☐ Once daily ☐ Twice weekly<br>☐ Twice daily ☐ PRN<br>☐ Once weekly ☐ Other (Please specify)	☐☐☐
Corticosteroid	☐	☐	☐ Oral ☐ Subcutaneous<br>☐ IV ☐ Nasal<br>☐ IM ☐ Topical<br>☐ Other (Please specify)	☐ Tablet<br>☐ Sachet<br>☐ Vial<br>☐ Other (Please specify)	☐ Once daily ☐ Twice weekly<br>☐ Twice daily ☐ PRN<br>☐ Once weekly ☐ Other (Please specify)	☐☐☐

Participant ID: ☐☐☐☐

Drug	Yes	No	Route of Administration	Quantity Unit	Frequency	Total Quantity Prescribed
Cytotoxic	☐	☐	☐ Oral ☐ Subcutaneous ☐ IV ☐ Nasal ☐ IM ☐ Topical ☐ Other (Please specify) ____	☐ Tablet ☐ Sachet ☐ Vial ☐ Other (Please specify) ____	☐ Once daily ☐ Twice weekly ☐ Twice daily ☐ PRN ☐ Once weekly ☐ Other (Please specify) ____	☐☐☐
Immunosuppressant	☐	☐	☐ Oral ☐ Subcutaneous ☐ IV ☐ Nasal ☐ IM ☐ Topical ☐ Other (Please specify) ____	☐ Tablet ☐ Sachet ☐ Vial ☐ Other (Please specify) ____	☐ Once daily ☐ Twice weekly ☐ Twice daily ☐ PRN ☐ Once weekly ☐ Other (Please specify) ____	☐☐☐
NSAID	☐	☐	☐ Oral ☐ Subcutaneous ☐ IV ☐ Nasal ☐ IM ☐ Topical ☐ Other (Please specify) ____	☐ Tablet ☐ Sachet ☐ Vial ☐ Other (Please specify) ____	☐ Once daily ☐ Twice weekly ☐ Twice daily ☐ PRN ☐ Once weekly ☐ Other (Please specify) ____	☐☐☐
Vasodilator	☐	☐	☐ Oral ☐ Subcutaneous ☐ IV ☐ Nasal ☐ IM ☐ Topical ☐ Other (Please specify) ____	☐ Tablet ☐ Sachet ☐ Vial ☐ Other (Please specify) ____	☐ Once daily ☐ Twice weekly ☐ Twice daily ☐ PRN ☐ Once weekly ☐ Other (Please specify) ____	☐☐☐

Participant ID: ☐☐☐☐

Drug	Yes	No	Route of Administration	Quantity Unit	Frequency	Total Quantity Prescribed
Other (please specify) ☐	☐	☐	☐ Oral ☐ Subcutaneous ☐ IV ☐ Nasal ☐ IM ☐ Topical ☐ Other (Please specify)	☐ Tablet ☐ Sachet ☐ Vial ☐ Other (Please specify)	☐ Once daily ☐ Twice weekly ☐ Twice daily ☐ PRN ☐ Once weekly ☐ Other (Please specify)	☐☐☐
Other (please specify) ☐	☐	☐	☐ Oral ☐ Subcutaneous ☐ IV ☐ Nasal ☐ IM ☐ Topical ☐ Other (Please specify)	☐ Tablet ☐ Sachet ☐ Vial ☐ Other (Please specify)	☐ Once daily ☐ Twice weekly ☐ Twice daily ☐ PRN ☐ Once weekly ☐ Other (Please specify)	☐☐☐
Other (please specify) ☐	☐	☐	☐ Oral ☐ Subcutaneous ☐ IV ☐ Nasal ☐ IM ☐ Topical ☐ Other (Please specify)	☐ Tablet ☐ Sachet ☐ Vial ☐ Other (Please specify)	☐ Once daily ☐ Twice weekly ☐ Twice daily ☐ PRN ☐ Once weekly ☐ Other (Please specify)	☐☐☐
Other(please specify) ☐	☐	☐	☐ Oral ☐ Subcutaneous ☐ IV ☐ Nasal ☐ IM ☐ Topical ☐ Other (Please specify)	☐ Tablet ☐ Sachet ☐ Vial ☐ Other (Please specify)	☐ Once daily ☐ Twice weekly ☐ Twice daily ☐ PRN ☐ Once weekly ☐ Other (Please specify)	☐☐☐

Please proceed to Section C

Participant ID: ☐☐☐☐

Section C - Inpatient Admissions

Has the participant had any inpatient admissions in the past 12 months in relation to their wound? ☐ Yes ☐ No

If Yes, please complete the information below for each admission.

If No, please proceed to section D.

Admission Date	Surgical Intervention Provided	Other Treatment Provided	Discharge Date	Adverse Event
☐☐ / ☐☐ / ☐☐☐☐<br>Day Month Year	Yes ☐ No ☐<br>If Yes, please tick all that apply:<br>Amputation ☐ Angloplasty ☐<br>Incision & Drainage ☐ Debridement ☐<br>Revascularisation ☐ Wound Closure ☐<br>☐ Other, please specify	Yes ☐ No ☐<br>If Yes, please tick all that apply:<br>Analgesia ☐ Antibiotic ☐<br>Larvae ☐ Topical Treatments ☐<br>Wound Management Systems ☐<br>☐ Other, please specify	☐☐ / ☐☐ / ☐☐☐☐<br>Day Month Year	Reported?<br>Yes ☐ No ☐<br>AE Number<br>☐☐
☐☐ / ☐☐ / ☐☐☐☐<br>Day Month Year	Yes ☐ No ☐<br>If Yes, please tick all that apply:<br>Amputation ☐ Angloplasty ☐<br>Incision & Drainage ☐ Debridement ☐<br>Revascularisation ☐ Wound Closure ☐<br>☐ Other, please specify	Yes ☐ No ☐<br>If Yes, please tick all that apply:<br>Analgesia ☐ Antibiotic ☐<br>Larvae ☐ Topical Treatments ☐<br>Wound Management Systems ☐<br>☐ Other, please specify	☐☐ / ☐☐ / ☐☐☐☐<br>Day Month Year	Reported?<br>Yes ☐ No ☐<br>AE Number<br>☐☐

Participant ID: ☐☐☐☐

Admission Date	Surgical Intervention Provided	Other Treatment Provided	Discharge Date	Adverse Event
☐☐ / ☐☐ / ☐☐☐☐<br>*Day Month Year*	Yes ☐ No ☐<br>If Yes, please tick all that apply:<br>Amputation ☐ Angloplasty ☐<br>Incision & Drainage ☐ Debridement ☐<br>Revascularisation ☐ Wound Closure ☐<br>☐ Other, please specify	Yes ☐ No ☐<br>If Yes, please tick all that apply:<br>Analgesia ☐ Antibiotic ☐<br>Larvae ☐ Topical Treatments ☐<br>Wound Management Systems ☐<br>☐ Other, please specify	☐☐ / ☐☐ / ☐☐☐☐<br>*Day Month Year*	Reported?<br>Yes ☐ No ☐<br>AE Number<br>☐☐
☐☐ / ☐☐ / ☐☐☐☐<br>*Day Month Year*	Yes ☐ No ☐<br>If Yes, please tick all that apply:<br>Amputation ☐ Angloplasty ☐<br>Incision & Drainage ☐ Debridement ☐<br>Revascularisation ☐ Wound Closure ☐<br>☐ Other, please specify	Yes ☐ No ☐<br>If Yes, please tick all that apply:<br>Analgesia ☐ Antibiotic ☐<br>Larvae ☐ Topical Treatments ☐<br>Wound Management Systems ☐<br>☐ Other, please specify	☐☐ / ☐☐ / ☐☐☐☐<br>*Day Month Year*	Reported?<br>Yes ☐ No ☐<br>AE Number<br>☐☐
☐☐ / ☐☐ / ☐☐☐☐<br>*Day Month Year*	Yes ☐ No ☐<br>If Yes, please tick all that apply:<br>Amputation ☐ Angloplasty ☐<br>Incision & Drainage ☐ Debridement ☐<br>Revascularisation ☐ Wound Closure ☐<br>☐ Other, please specify	Yes ☐ No ☐<br>If Yes, please tick all that apply:<br>Analgesia ☐ Antibiotic ☐<br>Larvae ☐ Topical Treatments ☐<br>Wound Management Systems ☐<br>☐ Other, please specify	☐☐ / ☐☐ / ☐☐☐☐<br>*Day Month Year*	Reported?<br>Yes ☐ No ☐<br>AE Number<br>☐☐

Participant ID: ☐☐☐☐

Admission Date	Surgical Intervention Provided	Other Treatment Provided	Discharge Date	Adverse Event
☐☐ / ☐☐ / ☐☐☐☐<br>*Day Month Year*	Yes ☐ No ☐<br>If Yes, please tick all that apply:<br>Amputation ☐ Angloplasty ☐<br>Incision & Drainage ☐ Debridement ☐<br>Revascularisation ☐ Wound Closure ☐<br>☐ Other, please specify	Yes ☐ No ☐<br>If Yes, please tick all that apply:<br>Analgesia ☐ Antibiotic ☐<br>Larvae ☐ Topical Treatments ☐<br>Wound Management Systems ☐<br>☐ Other, please specify	☐☐ / ☐☐ / ☐☐☐☐<br>*Day Month Year*	Reported?<br>Yes ☐ No ☐<br>AE Number<br>☐☐
☐☐ / ☐☐ / ☐☐☐☐<br>*Day Month Year*	Yes ☐ No ☐<br>If Yes, please tick all that apply:<br>Amputation ☐ Angloplasty ☐<br>Incision & Drainage ☐ Debridement ☐<br>Revascularisation ☐ Wound Closure ☐<br>☐ Other, please specify	Yes ☐ No ☐<br>If Yes, please tick all that apply:<br>Analgesia ☐ Antibiotic ☐<br>Larvae ☐ Topical Treatments ☐<br>Wound Management Systems ☐<br>☐ Other, please specify	☐☐ / ☐☐ / ☐☐☐☐<br>*Day Month Year*	Reported?<br>Yes ☐ No ☐<br>AE Number<br>☐☐

Please provide details of any additional visits using the SWHSI-2 Supplementary Page for Inpatient Admissions.

Please proceed to Section D

Participant ID: ☐☐☐☐

Section D - Outpatient Visits

Has the participant had any outpatient visits in the past 12 months in relation to their wound? ☐ Yes ☐ No

If Yes, please complete the questions below for each outpatient visit.

If No, please proceed to section E.

Visit Date	Clinic Type	Other Treatment Provided	Adverse Event
☐☐ / ☐☐ / ☐☐☐☐<br>Day Month Year	Diabetic Foot Clinic ☐ Occupational Therapy ☐<br>Pain Management ☐ Physiotherapy ☐<br>Podiatry ☐ Post Surgical Review ☐<br>Speciality Dressing Clinic ☐ Tissue Viability ☐<br>Other, please specify ☐	Yes ☐ No ☐<br>If Yes, please tick all that apply:<br>Larvae ☐ Analgesia ☐ Antibiotic ☐<br>Dietetic Input ☐ Topical Treatments ☐<br>Wound Management Systems ☐<br>Other, please specify ☐	Reported?<br>Yes ☐ No ☐<br>AE Number<br>☐☐
☐☐ / ☐☐ / ☐☐☐☐<br>Day Month Year	Diabetic Foot Clinic ☐ Occupational Therapy ☐<br>Pain Management ☐ Physiotherapy ☐<br>Podiatry ☐ Post Surgical Review ☐<br>Speciality Dressing Clinic ☐ Tissue Viability ☐<br>Other, please specify ☐	Yes ☐ No ☐<br>If Yes, please tick all that apply:<br>Larvae ☐ Analgesia ☐ Antibiotic ☐<br>Dietetic Input ☐ Topical Treatments ☐<br>Wound Management Systems ☐<br>Other, please specify ☐	Reported?<br>Yes ☐ No ☐<br>AE Number<br>☐☐

Participant ID: ☐☐☐☐

Visit Date	Clinic Type	Other Treatment Provided	Adverse Event
☐☐ / ☐☐ / ☐☐☐☐<br>*Day* *Month* *Year*	Diabetic Foot Clinic ☐ Occupational Therapy ☐<br>Pain Management ☐ Physiotherapy ☐<br>Podiatry ☐ Post Surgical Review ☐<br>Speciality Dressing Clinic ☐ Tissue Viability ☐<br>Other, please specify ☐	Yes ☐ No ☐<br>If Yes, please tick all that apply:<br>Larvae ☐ Analgesia ☐ Antibiotic ☐<br>Dietetic Input ☐ Topical Treatments ☐<br>Wound Management Systems ☐<br>Other, please specify ☐	Reported?<br>Yes ☐ No ☐<br>AE Number<br>☐☐
☐☐ / ☐☐ / ☐☐☐☐<br>*Day* *Month* *Year*	Diabetic Foot Clinic ☐ Occupational Therapy ☐<br>Pain Management ☐ Physiotherapy ☐<br>Podiatry ☐ Post Surgical Review ☐<br>Speciality Dressing Clinic ☐ Tissue Viability ☐<br>Other, please specify ☐	Yes ☐ No ☐<br>If Yes, please tick all that apply:<br>Larvae ☐ Analgesia ☐ Antibiotic ☐<br>Dietetic Input ☐ Topical Treatments ☐<br>Wound Management Systems ☐<br>Other, please specify ☐	Reported?<br>Yes ☐ No ☐<br>AE Number<br>☐☐
☐☐ / ☐☐ / ☐☐☐☐<br>*Day* *Month* *Year*	Diabetic Foot Clinic ☐ Occupational Therapy ☐<br>Pain Management ☐ Physiotherapy ☐<br>Podiatry ☐ Post Surgical Review ☐<br>Speciality Dressing Clinic ☐ Tissue Viability ☐<br>Other, please specify ☐	Yes ☐ No ☐<br>If Yes, please tick all that apply:<br>Larvae ☐ Analgesia ☐ Antibiotic ☐<br>Dietetic Input ☐ Topical Treatments ☐<br>Wound Management Systems ☐<br>Other, please specify ☐	Reported?<br>Yes ☐ No ☐<br>AE Number<br>☐☐

Participant ID: ☐☐☐☐

Visit Date	Clinic Type	Other Treatment Provided	Adverse Event
☐☐ / ☐☐ / ☐☐☐☐<br>*Day* *Month* *Year*	Diabetic Foot Clinic ☐ Occupational Therapy ☐<br>Pain Management ☐ Physiotherapy ☐<br>Podiatry ☐ Post Surgical Review ☐<br>Speciality Dressing Clinic ☐ Tissue Viability ☐<br>Other, please specify ☐	Yes ☐ No ☐<br>If Yes, please tick all that apply:<br>Larvae ☐ Analgesia ☐ Antibiotic ☐<br>Dietetic Input ☐ Topical Treatments ☐<br>Wound Management Systems ☐<br>Other, please specify ☐	Reported?<br>Yes ☐ No ☐<br>AE Number<br>☐☐
☐☐ / ☐☐ / ☐☐☐☐<br>*Day* *Month* *Year*	Diabetic Foot Clinic ☐ Occupational Therapy ☐<br>Pain Management ☐ Physiotherapy ☐<br>Podiatry ☐ Post Surgical Review ☐<br>Speciality Dressing Clinic ☐ Tissue Viability ☐<br>Other, please specify ☐	Yes ☐ No ☐<br>If Yes, please tick all that apply:<br>Larvae ☐ Analgesia ☐ Antibiotic ☐<br>Dietetic Input ☐ Topical Treatments ☐<br>Wound Management Systems ☐<br>Other, please specify ☐	Reported?<br>Yes ☐ No ☐<br>AE Number<br>☐☐

Please provide details of any additional visits using the SWHSI-2 Supplementary Page for Outpatient Visits.

Please proceed to Section E

Participant ID: ☐☐☐☐

Section E - Accident & Emergency Visits

Has the participant had any Accident & Emergency visits in the past 12 months in relation to their wound? ☐ Yes ☐ No

If Yes, please complete the questions below for each Accident & Emergency visit. Please tick all interventions provided at each visit.

If No, please proceed to Section F.

Visit Date	Intervention provided	Was the patient admitted as an inpatient following the visit?	Adverse Event
☐☐ / ☐☐ / ☐☐☐☐ *Day Month Year*	☐ Antibiotic ☐ Analgesia ☐ Larvae<br>☐ Topical Treatments<br>☐ Wound Management Systems<br>☐ Other, please specify	Yes ☐ No ☐	Reported?<br>Yes ☐ No ☐<br>AE Number ☐☐
☐☐ / ☐☐ / ☐☐☐☐ *Day Month Year*	☐ Antibiotic ☐ Analgesia ☐ Larvae<br>☐ Topical Treatments<br>☐ Wound Management Systems<br>☐ Other, please specify	Yes ☐ No ☐	Reported?<br>Yes ☐ No ☐<br>AE Number ☐☐
☐☐ / ☐☐ / ☐☐☐☐ *Day Month Year*	☐ Antibiotic ☐ Analgesia ☐ Larvae<br>☐ Topical Treatments<br>☐ Wound Management Systems<br>☐ Other, please specify	Yes ☐ No ☐	Reported?<br>Yes ☐ No ☐<br>AE Number ☐☐

Participant ID: ☐☐☐☐

Visit Date	Intervention provided	Was the patient admitted as an inpatient following the visit?	Adverse Event
☐☐ / ☐☐ / ☐☐☐☐<br>*Day* *Month* *Year*	☐ Antibiotic ☐ Analgesia ☐ Larvae<br>☐ Topical Treatments<br>☐ Wound Management Systems<br>☐ Other, please specify	Yes ☐ No ☐	Reported?<br>Yes ☐ No ☐<br>AE Number ☐☐
☐☐ / ☐☐ / ☐☐☐☐<br>*Day* *Month* *Year*	☐ Antibiotic ☐ Analgesia ☐ Larvae<br>☐ Topical Treatments<br>☐ Wound Management Systems<br>☐ Other, please specify	Yes ☐ No ☐	Reported?<br>Yes ☐ No ☐<br>AE Number ☐☐
☐☐ / ☐☐ / ☐☐☐☐<br>*Day* *Month* *Year*	☐ Antibiotic ☐ Analgesia ☐ Larvae<br>☐ Topical Treatments<br>☐ Wound Management Systems<br>☐ Other, please specify	Yes ☐ No ☐	Reported?<br>Yes ☐ No ☐<br>AE Number ☐☐
☐☐ / ☐☐ / ☐☐☐☐<br>*Day* *Month* *Year*	☐ Antibiotic ☐ Analgesia ☐ Larvae<br>☐ Topical Treatments<br>☐ Wound Management Systems<br>☐ Other, please specify	Yes ☐ No ☐	Reported?<br>Yes ☐ No ☐<br>AE Number ☐☐

Please provide details of any additional visits using the SWHSI-2 Supplementary Page for A&E Visits. *Please proceed to Section F.*

Participant ID: ☐☐☐☐

Section F - Hospital Transport

During the past 12 months, has the participant required any use of hospital transport in relation to their wound? ☐ Yes ☐ No

If Yes, please indicate the total number of times transport has been required: ☐☐☐

Please proceed to Section G

Section G - CRF Sign Off

	Yes	No
All sections of the Investigator CRF have been completed as required	☐	☐

Form Completed by:

*Name: ____________________ Signature: ____________________

Assessor ID: ☐☐ Date: ☐☐ / ☐☐ / 2 0 ☐☐
Day Month Year

*Must be reflected in the Delegation of Authority Log
