# Supplementary material for: Negative pressure wound therapy versus usual care for surgical wounds healing by secondary intention (SWHSI-2 trial): study protocol for a pragmatic, multicentre, cross surgical specialty, randomised controlled trial
Source: Trials. 2021 Oct 25;22:739. doi: 10.1186/s13063-021-05662-2 (PMC8543414; doi:10.1186/s13063-021-05662-2)
Supplement: Supplementary file 14 — Additional file 14. Baseline Investigator Case Report Form [file 13063_2021_5662_MOESM14_ESM.pdf]

The Department of Health Sciences

Hull University Teaching Hospitals NHS Trust

SWHSI-2 Trial

Baseline Investigator Case Report Form

FOR STUDY INVESTIGATOR COMPLETION

Site ID: ☐☐

Participant ID: ☐☐☐☐

Assessment Date: ☐☐ / ☐☐ / ☐☐☐☐
day *month* *year*

This project is funded by the National Institute for Health Research Health Technology Assessment Programme (Project number 17/42/94)

Section A – Patient Eligibility and Consent Check

Please complete the checklist below to confirm patient eligibility and consent form completion before proceeding to complete this case report form (CRF).

Note: Informed consent must be obtained prior to any trial procedures being undertaken, including completion of any section of this form.

1. Have all the inclusion criteria been met? ☐ Yes ☐ No
2. Are any of the exclusion criteria present? ☐ Yes ☐ No
3. Has the patient provided written informed consent? ☐ Yes ☐ No

<u>If the patient is eligible and consent has been provided, please read the instructions for completing this CRF</u>:

This CRF may be completed by the principal investigator or a delegated member of staff listed on the SWHSI-2 Trial Delegation Log.

Please refer to the associated SWHSI-2 Trial Specific Procedure for full details of how to complete this CRF.

Please complete all sections of this questionnaire using the spaces provided, and sign off when complete.

Please do not include any patient identifiable information when completing this CRF. When complete, please remove the staple and take a photocopy of the completed CRF for your site records. **Please do not re-staple the original.** Place the unstapled original in a "SWHSI-2 Trial business reply envelope" and send via post to York Trials Unit.

Please proceed to Section B

Section B – Patient demographics

1. Date of Birth ☐☐ / ☐☐ / ☐☐☐☐
   day *month* *year*

2. Gender ☐ Male ☐ Female

3. Ethnicity *(please cross one box only)*

   ☐ White ☐ Mixed race ☐ Asian or Asian British

   ☐ Black or Black British ☐ Chinese

   ☐ Other ethnicity, please specify: ____________

4. Smoking status
   - Tobacco ☐ Never ☐ Current ☐ Previous
   - E-cigarette ☐ Never ☐ Current ☐ Previous

5. Alcohol intake: ☐ Yes ☐ No

   If 'Yes' Average Units per week: ☐☐☐ units

Please proceed to Section C

Section C – Comorbidities

Does the patient currently have any of the following co-morbidities? ☐ Yes ☐ No

If 'Yes', please complete Questions 1 to 6. If 'No', please proceed to Section D.

---

1. Cardiovascular disease: ☐ Yes ☐ No

   If 'Yes' please specify:

   ☐ Hypertension ☐ Myocardial Infarction ☐ Angina ☐ Heart Failure

   ☐ Other, please specify: ____________________

---

2. Peripheral Vascular Disease: ☐ Yes ☐ No

   If 'Yes' please specify:

   ☐ Asymptomatic ☐ Claudication ☐ Rest Pain ☐ Tissue Loss ☐ Aneurysm

   ☐ Vasculitis ☐ Other, please specify: ____________________

---

3. Diabetes: ☐ Yes ☐ No

   If 'Yes' is this controlled by:

   ☐ Insulin ☐ Tablet ☐ Diet

---

4. Respiratory conditions: ☐ Yes ☐ No

---

5. Neurological conditions: ☐ Yes ☐ No

   If 'Yes' please specify:

   ☐ Parkinson's Disease ☐ Multiple Sclerosis ☐ Epilepsy ☐ Spinal injury

   ☐ Other, please specify: ____________________

---

6. Stroke: ☐ Yes ☐ No

Please proceed to Section D

Section D – Concomitant Medications

Is the patient currently receiving any of the following medications? ☐ Yes ☐ No

If ‘Yes’, provide a response for all listed below. If 'No', please proceed to Section E.

Cytotoxic	☐ Yes	☐ No
NSAID	☐ Yes	☐ No
Anti-coagulant	☐ Yes	☐ No
Antiplatelet	☐ Yes	☐ No
Corticosteroid	☐ Yes	☐ No
Immunosuppressant	☐ Yes	☐ No
Vasodilator	☐ Yes	☐ No
Analgesia	☐ Yes	☐ No

If ‘Yes’, please indicate the type and reason:

☐ IV ☐ Oral ☐ Other: ________

☐ Related to SWHSI ☐ Other reason: ________

Antibiotic	☐ Yes	☐ No

If ‘Yes’, please indicate the type and reason:

☐ IV ☐ Oral ☐ Other: ________

☐ Related to SWHSI ☐ Other reason: ________

Please proceed to Section E

Section E – Body Mass Index Measurements

Please record the patients:

Height: ☐☐☐ *cm* Weight: ☐☐☐.☐☐ *Kgs*

Please proceed to Section F

Section F – Surgery Details

1. Please record the type of surgery that led to this SWHSI:

☐ Vascular ☐ Colorectal ☐ Plastics
☐ Other (please specify): ____________

2. Please record the date of the surgery that led to the SWHSI: ☐☐ / ☐☐ / ☐☐☐☐
day *month* *year*

3. Was the surgery: ☐ Elective ☐ Emergency

4. Please record the name of the surgery:

☐ Laparotomy with bowel resections
☐ Laparotomy without bowel resections
☐ Excision of pilonidal sinus
☐ Hernia repair
☐ Minor lower limb amputation
☐ Major lower limb amputation
☐ Cholecystectomy
☐ Hysterectomy
☐ Caesarean Section
☐ Excisions of skin lesions
☐ Surgical debridement of necrotic tissue
☐ Incision and drainage of abscess (including related to diabetic foot)
☐ Other (please specify): ____________

5. The contamination level of surgery was:

☐ Clean *(i.e. uninfected wound with no inflammation, and where respiratory, alimentary, genital or urinary tracts are not entered)*
☐ Clean Contaminated *(i.e. wound in which respiratory, alimentary, genital or urinary tracts are entered under controlled conditions and without contamination)*
☐ Contaminated *(i.e. open, fresh or accidental wounds, operations where a major break in sterile technique or gross spillage from intestinal tract, or where acute non-purulent inflammation is present)*
☐ Dirty *(i.e. wound with devitalised tissue, clinical infection or perforated viscera)*

6. Did the surgery involve a surgical implant? ☐ Yes ☐ No

If 'Yes', please provide details: ☐ Mesh ☐ Prosthesis ☐ Stent

☐ Other (please specify): ____________

Please proceed to Section G

Section G – SWHSI Assessment

1. How many SWHSI does this patient have?

☐ 1 ☐ 2 ☐ 3 ☐ 4 ☐ 5 ☐ 6+

If the patient has more than one SWHSI, the largest wound will be defined as the reference SWHSI.

2. Does this patient have a previous history of SWHSI? ☐ Yes ☐ No

3. The SWHSI arose because *(please select all that apply):*

☐ The wound edges could not be approximated

☐ Of presence of infection/contamination

☐ Planned to heal by secondary intention for other reasons, please specify:

____

☐ Wound dehisced along its entire length. If 'Yes', please provide the date this occurred:

☐☐ / ☐☐ / ☐☐☐☐
day *month* *year*

☐ Wound dehisced along part of its length. If 'Yes', please provide the date this occurred:

☐☐ / ☐☐ / ☐☐☐☐
day *month* *year*

☐ Previously closed wound surgically opened. If 'Yes', please provide the date this occurred:

☐☐ / ☐☐ / ☐☐☐☐
day *month* *year*

4. In your clinical opinion is the SWHSI currently infected? ☐ Yes ☐ No

If 'Yes', please indicate which features are present. If 'No', please proceed to Question 5.

☐ Abscess or other evidence of infection (found during re-operation, by radiology or histopath examination)

☐ Aspirated fluid swab yields organisms and pus cells are present

☐ Antibiotics prescribed for infection

☐ Incision spontaneously dehisces or was opened by a surgeon

☐ Clinician diagnosis

☐ Purulent drainage

☐ Fever

☐ Heat

☐ Localised swelling

☐ Localised pain and tenderness

☐ Redness

5. Please record if the wound has any of the following features:

Odour	☐ Yes	☐ No
Slough	☐ Yes	☐ No
Necrosis	☐ Yes	☐ No
Granulation Tissue	☐ Yes	☐ No
Exudate	☐ Yes	☐ No

If 'Yes', please indicate if this is: ☐ Low ☐ Medium ☐ High

Other (please specify): ☐ Yes ☐ No

6. Please grade the SWHSI in terms of tissue involvement: *(please select all that apply)*

- ☐ Full thickness skin loss
- ☐ Subcutaneous tissue exposed
- ☐ Underlying muscle exposed
- ☐ Underlying tendons exposed
- ☐ Underlying bone exposed
- ☐ Underlying organs exposed
- ☐ Tissue involvement unclear due to undermining or devitalised tissue
- ☐ Other (please specify):

7. Please record the wound size:

Wound Length: ☐☐☐.☐☐ *cm*

Wound Width: ☐☐☐.☐☐ *cm*

Wound Area: ☐☐☐.☐☐ *cm²*

8. If the patient’s wound is located on their foot, please indicate the patients status relating to the following features:

Pedal blood flow: ☐ Intact ☐ Reduced

Neuropathy: ☐ Protective sensation intact ☐ Protective sensation reduced ☐ Protective sensation absent

Please proceed to Section H

Section H – SWHSI Treatments

1. Where is the patient currently receiving treatment for their SWHSI?

- ☐ Hospital Outpatient Department
- ☐ Hospital Ward
- ☐ Community Hospital
- ☐ Intermediate Care Facility
- ☐ Podiatry Clinic
- ☐ GP Practice
- ☐ Own Home
- ☐ Another's Home
- ☐ Nursing Home
- ☐ Community Clinic
- ☐ Other (please specify): ______

2. Who is primarily responsible for the patient's care? *(please select one option only)*

- ☐ Hospital doctor
- ☐ General Practitioner
- ☐ Ward Nurse
- ☐ Tissue Viability Nurse
- ☐ Practice Nurse
- ☐ District Nurse
- ☐ Other (please specify): ______

3. Is the patient is currently receiving dressings for their SWHSI? ☐ Yes ☐ No

If 'Yes', please provide details of the dressings used:

- ☐ Alginate
- ☐ Basic wound contact
- ☐ Cavity Foam
- ☐ Foam
- ☐ Gauze Ribbon
- ☐ Hydrocolloid
- ☐ Hydrogel
- ☐ Hydrofoam/Spun Hydrocolloid
- ☐ Honey
- ☐ Iodine Containing
- ☐ PHMB
- ☐ Protease Modulating matrix
- ☐ Silver containing
- ☐ Flamazine silver sulfadiazine dressing
- ☐ Urgotol silver sulfadiazine dressing
- ☐ Soft polymer
- ☐ Superabsorbent
- ☐ Vapour permeable film or membrane
- ☐ Negative Pressure Wound Therapy* **Note patient should not be randomised if already receiving NPWT for their SWHSI*
- ☐ Other (please specify): ______
- ☐ Other (please specify): ______
- ☐ Other (please specify): ______

4. Is the patient receiving any other treatment for their SWHSI (excluding antibiotics and analgesia reported in Section D)? ☐ Yes ☐ No

If 'Yes', please provide details of the treatments used:

☐ Wound Management Systems ☐ Emollient ☐ Silver Nitrate Stick

☐ Dietetic Input ☐ Larvae

☐ Other (please specify): ____________________

☐ Other (please specify): ____________________

☐ Other (please specify): ____________________

Please proceed to Section I

Section I – Checklist and CRF Sign Off

1. All Sections of the Investigator CRF are complete ☐ Yes ☐ No
2. All Sections of the Participant CRF are complete ☐ Yes ☐ No
3. Wound measurements have been completed ☐ Yes ☐ No
4. Wound photograph has been taken ☐ Yes ☐ No

If 'No', please indicate why a photograph was not obtained:

____

Form Completed by:

*Name: ____________________

Signature: ____________________

Assessor ID: [ ][ ]

Date: [ ][ ] / [ ][ ] / [ ][ ][ ][ ]
day *month* *year*

*Must be reflected in the Delegation of Duties Log

Please proceed to Section J

Section J – Randomisation

Date of Randomisation: ☐☐ / ☐☐ / ☐☐☐☐
day *month* *year*

Randomised to receive: ☐ NPWT ☐ Usual Care
