# Supplementary material for: Negative pressure wound therapy versus usual care for surgical wounds healing by secondary intention (SWHSI-2 trial): study protocol for a pragmatic, multicentre, cross surgical specialty, randomised controlled trial
Source: Trials. 2021 Oct 25;22:739. doi: 10.1186/s13063-021-05662-2 (PMC8543414; doi:10.1186/s13063-021-05662-2)
Supplement: Supplementary file 15 — Additional file 15. Screening for Eligibility Case Report Form [file 13063_2021_5662_MOESM15_ESM.pdf]

UNIVERSITY of York
The Department of Health Sciences

NHS
Hull University Teaching Hospitals
NHS Trust

SWHSI-2 Trial

Screening for Eligibility Case Report Form

FOR STUDY INVESTIGATOR COMPLETION

Site ID: ☐☐

Participant ID: ☐☐☐☐

Assessment Date: ☐☐ / ☐☐ / ☐☐☐☐
day *month* *year*

This project is funded by the National Institute for Health Research Health Technology Assessment Programme (Project number 17/42/94)

Instructions for this Case Report Form

This case report form (CRF) should be completed for all patients who are potentially eligible for the SWHSI-2 Trial meeting the inclusion criteria of having a **surgical wound healing by secondary intention, of equal to or less than six weeks in duration**.

Assessment of eligibility may be completed preoperatively (e.g. SWHSI is planned) or postoperatively (e.g. SWHSI identified following surgery).

Where assessment is completed preoperatively it may not be possible to complete all questions prior to surgery. Items denoted with an '+' may require completion once the operation is completed. You will therefore need to ensure you revisit and fully complete this CRF either in theatre or immediately following the operation. **The CRF must be fully completed before randomisation**.

Please refer to the associated SWHSI-2 Trial Specific Procedure for full details of how to complete this CRF.

Potentially eligible patients can be identified by any member of the local clinical team.

Eligibility must be confirmed by the SWHSI-2 Trial Principal Investigator at site, a delegated medic or a registered nurse (as listed on the SWHSI-2 Trial Delegation Log).

Please complete all sections of this questionnaire using the spaces provided, and sign off when complete.

Please do not include any patient identifiable information when completing this CRF.

When complete, please remove the staple and take a photocopy of the completed CRF for your site records. **Please do not re-staple the original**. Place the unstapled original in a "SWHSI-2 Trial business reply envelope" and send via post to York Trials Unit.

Participant ID: ☐☐☐☐

Section A – Suitability of Assessment

Screening assessment is being conducted ☐ Preoperatively ☐ Postoperatively

+**Potential participant has a SWHSI** *(defined as an open surgical wound not healing by primary intention but from the 'bottom up'*)*

☐ Yes ☐ No

***The following are NOT considered to be SWHSI in this study**

1. Wounds with planned delayed primary closure (e,g, open wound to be left open for 5 to 7 days then closured planned. If however, *if these wounds then convert to SWHSI (as defined by the inclusion criteria), they would become eligible for inclusion within this study).*
2. Wounds left open without planned healing *(e.g. stoma, tracheotomy, gastrostomy).*
3. Surgery that does not involve an incision on the skin surface *(e.g. tonsillectomy, dilation and curettage (i.e. "internal" wounds)).*
4. Split skin donor graft sites.
5. Nail avulsions.
6. Cavities resulting from dental extractions.
7. Operations involving the eye *(i.e. cataract surgery and removal of the eye).*
8. Wounds that are a consequence of minor dermatological or plastic surgery *(e.g. removal of warts, skin tags) or diagnostic procedures (e.g. punch biopsy).*
9. Chronic wounds such as pressure ulcers or foot ulcers that were non-surgical in origin.

+**Please indicate where the patient's SWHSI is primarily located:**
(Please cross ONE option only)

☐ Head ☐ Neck ☐ Arm ☐ Breast ☐ Hand

☐ Abdomen ☐ Natal Cleft ☐ Back ☐ Buttocks ☐ Leg

☐ Perineum/Peri-anal area ☐ Fore or midfoot ☐ Hindfoot

☐ Other please specify: ____________________

Please proceed to Section B

Section B - Eligibility Assessment

**Inclusion Criteria**	**Yes**	**No**
Patient is aged 16 years or over.	☐	☐
+Has an acute SWHSI *(i.e. a wound left open as planned following surgery or a wound initially closed using sutures, clips, or other closure methods and dehisced along the whole or part of their length, and of less than 6 weeks in duration)*, arising from any surgical specialty and occurring on any part of the body, deemed appropriate to receive either NPWT or wound dressing treatment.	☐	☐
+Patient has a SWHSI that is considered ready for negative pressure wound therapy (NPWT) treatment *i.e. contains at least 80% viable tissue or has only a thin layer of slough requiring no further debridement.*	☐	☐
Patient is able to give informed consent and provide follow up data.	☐	☐

**Exclusion Criteria**	**Yes**	**No**
Patient is deemed to be malnourished, as per NICE guidelines CG 32 (29) (BMI <18.5 kg/m² ; unplanned* weight loss >10% in the last 3-6 months; BMI <20kg/m² and unplanned* weight loss >5% in the last 3-6 months) or is assessed as at high risk of malnutrition using the Malnutrition Universal Screening Tool (MUST) (28).<br><br>**Patients with weight loss arising either from underlying comorbidity (e.g. ulcerative colitis) or from the reasons for surgery being completed (e.g. bowel cancer) may be included at the clinician's discretion.*	☐	☐
Patient has life expectancy of less than 6-months.	☐	☐
+Patient has active systemic infection as defined by clinical and/or laboratory assessment.<br><br>Note: If patient has an active infection but is improving following 1 weeks duration of antibiotics, please tick here to confirm that the patient is appropriate for inclusion as per clinical discretion: ☐	☐	☐
Patient is without adequate haemostasis and/or at risk of bleeding.	☐	☐
Wound is chronic and non-surgical in origin *(e.g. pressure ulcer, foot ulcer)*	☐	☐

Participant ID: ☐☐☐☐

Exclusion Criteria (continued)	Yes	No
+Patient is already receiving, or has previously received, NPWT on their wound.<br>**If 'Yes'**, the patient has received NPWT due to:<br>☐ Patient Preference<br>☐ Surgeon Preference<br>☐ Nurse Preference<br>☐ Applied before R&D approval in place<br>☐ Received in theatre<br>☐ Other (give reason): ____<br>☐ Reason Unknown	☐	☐
Delayed primary closure of the wound is planned.	☐	☐
+The patient or wound is contraindicated to NPWT<br>**If 'Yes'**, the patient or wound is contraindicated due to:<br>☐ Unclear undermining in the wound cavity (i.e. the deepest point of the wound cannot be measured)<br>☐ Necrotic tissue, malignant tissue or eschar are present<br>☐ Exposed blood vessels and/or organs, anastomotic sites and/or nerves are present<br>☐ Wound is situated where a vacuum seal cannot be obtained<br>☐ A non-enteric or unexplored fistula is present<br>☐ Patient requires emergency airway aspiration, pleural mediastinal or chest tube drainage or surgical suction	☐	☐
Patient has participated in another wound research study where the primary outcome time point has not yet been reached.	☐	☐

Please proceed to Section C

Section C – Eligibility Confirmation

The patient is eligible if

- ALL inclusion criteria are answered YES
- ALL exclusion criteria are answered NO

1. Is the patient eligible to participate in the trial, based on the inclusion and exclusion criteria?

☐ Yes ☐ No

If 'Yes', please proceed to Question 2.

If 'No', please proceed to Section E.

2. Will the patient be approached for consent? ☐ Yes ☐ No

If 'Yes', please provide the patient with an information sheet and proceed to Section D .

If 'No', please provide a reason in the box below and proceed to Section E
(Note: Please do not include any patient identifiable information when detailing reason)

Section D – Consent Confirmation

1. Has the patient provided written informed consent to take part in the SWHSI-2 trial?

☐ Yes ☐ No

(Note: No study activity can be undertaken unless informed consent is received)

If 'Yes', please proceed to Question 2.

If 'No', please give details of the reasons for patient non-consent:

☐ Patient unwilling to participate in research

☐ Patient unwilling to be randomised to a treatment

☐ Other reason (describe reasons in box below):

(Note: Please do not include any patient identifiable information when detailing reason)

☐ Not known

2. Does the patient express a preference for treatment?

☐ NPWT ☐ Usual Care ☐ No preference

Please proceed to Section E

Section E – Sign Off

Eligibility Confirmed By:

*Name:

Signature:

Assessor ID:

Date: / /

day month year

Form Completed by (if different to above):

*Name:

Signature:

Assessor ID:

Date: / /

day month year

*Must be reflected in the Delegation of Duties Log
