# Supplementary material for: Negative pressure wound therapy versus usual care for surgical wounds healing by secondary intention (SWHSI-2 trial): study protocol for a pragmatic, multicentre, cross surgical specialty, randomised controlled trial
Source: Trials. 2021 Oct 25;22:739. doi: 10.1186/s13063-021-05662-2 (PMC8543414; doi:10.1186/s13063-021-05662-2)
Supplement: Supplementary file 16 — Additional file 16. SWHSI-2 Participant Change of Status Form [file 13063_2021_5662_MOESM16_ESM.pdf]

SWHSI-2 Participant Change of Status Form

Study Title: SWHSI-2			
REC Reference:	19/YH/0054	ISRCTN number:	
Sponsor:	Hull and East Yorkshire Hospitals NHS Trust	Chief Investigator:	Professor Ian Chetter

Please complete this form when there is a change in the status of a participant.

If the patient requests full withdrawal during a study assessment, please ask the participant if they would be willing to complete the current study assessment questionnaires. If they are willing, please complete this form once all questionnaires are completed for this assessment.

If the patient requests full withdrawal between study assessment, complete and date this form when the participant first requested withdrawal.

1. Participant details

Participant study number: ☐☐☐☐ Site ID: ☐☐

2. Timing of change

Change in status date: ☐☐ / ☐☐ / ☐☐☐☐
day month year

Did the participant receive their allocated treatment prior to status change? ☐ Yes ☐ No

3. Please indicate whether the participant died OR changed follow up status and provide details at the end of the form.

☐ **Participant has died** Date of death: ☐☐ / ☐☐ / ☐☐☐☐
day month year

A Serious Adverse Event form has been completed: ☐ Yes ☐ No*

*If 'No', please complete a Serious Adverse Event form and send to York Trials Unit

☐ **Participant follow up status has changed**

a) Type of status change
Please indicate the participant new status below. Note that the telephone data collection is intended to continue only until wound healing.

☐ Participant is to be followed up by postal questionnaire only (i.e. patient is withdrawing from nurse assessment (telephone or healing follow up)

If 'Yes', is the participant willing to be contacted by telephone by the research nurse for collection of adverse event data only ☐ Yes ☐ No

☐ Participant is to be followed up by nurse assessment only (i.e. the participant is withdrawing from postal follow up)

☐ Participant is being fully withdrawn

b) *Continued data collection*
(Complete only if the participant has withdrawn from nurse assessment, or has withdrawn in full)

Does the participant agree to the research team obtaining data from healthcare professionals? ☐ Yes ☐ No

c) *Data retention*
(Complete only if the participant has withdrawn in full)

Does the participant agree to continued storage of personal details? ☐ Yes ☐ No

4. Please provide further information for change in status:

Name of person completing form (if different to PI): ____________________

Signature of person completing form: ____________________

Assessor ID: ☐☐ **Date:** ☐☐ / ☐☐ / ☐☐☐☐
day *month* *year*

CONFIRMED BY PI/DELEGATED CLINICIAN

Name: ____________________

Signature: ____________________

Assessor ID: ☐☐ **Date:** ☐☐ / ☐☐ / ☐☐☐☐
day *month* *year*

Please send a copy of the form to York Trials Unit either by fax: 01904 321387 or email: swhsi2-trial-group@york.ac.uk and file the original form in your site files.
