# Supplementary material for: Negative pressure wound therapy versus usual care for surgical wounds healing by secondary intention (SWHSI-2 trial): study protocol for a pragmatic, multicentre, cross surgical specialty, randomised controlled trial
Source: Trials. 2021 Oct 25;22:739. doi: 10.1186/s13063-021-05662-2 (PMC8543414; doi:10.1186/s13063-021-05662-2)
Supplement: Supplementary file 17 — Additional file 17. SWHSI-2 Blinded Outcome Assessment CRF [file 13063_2021_5662_MOESM17_ESM.pdf]

SWHSI-2 Blinded Outcome Assessment CRF

Participant ID: ☐☐☐☐ Date of Blinded Assessment: ☐☐ / ☐☐ / ☐☐☐☐
day month year

Assessment completed by: ☐ LW ☐ AO ☐ NS ☐ Other, please specify ☐☐

Please complete both Questions 1 and 2 when reviewing SWHSI-2 Photographs.

1. Do the photographs taken for this participant confirm reference wound healing?
   (Please select one option)

   ☐ **Yes**

   ☐ **No** If 'No', please provide information regarding why you suggest this wound is not healed:

   ☐ **Unsure** If 'Unsure', please indicate reasons for uncertainty:

   - ☐ Photograph is blurred or out of focus
   - ☐ Wound is not fully visible in photograph
   - ☐ Photograph taken too far away from wound
   - ☐ Photograph is too dark
   - ☐ Other, please specify:
   - ☐ Other, please specify:

   Do you have any notes or comments, including other reasons for uncertainty?

   ☐ Yes ☐ No

   If 'Yes', please provided these below:

2. Do you think that you know what treatment the participant received?

   ☐ **Yes** ☐ **No**

   If 'Yes', please indicate the treatment you believe the participant received:

   ☐ Intervention – Negative Pressure Wound Therapy

   ☐ Usual Care – Wound Dressings

REC Reference: 19/YH/0054
Sponsor Reference: R2319
IRAS Reference: 258802

4266471549
