# Supplementary material for: Negative pressure wound therapy versus usual care for surgical wounds healing by secondary intention (SWHSI-2 trial): study protocol for a pragmatic, multicentre, cross surgical specialty, randomised controlled trial
Source: Trials. 2021 Oct 25;22:739. doi: 10.1186/s13063-021-05662-2 (PMC8543414; doi:10.1186/s13063-021-05662-2)
Supplement: Supplementary file 19 — Additional file 19. PARTICIPANT CONSENT FORM [file 13063_2021_5662_MOESM19_ESM.pdf]

INSERT TRUST LOGO

PARTICIPANT CONSENT FORM

Surgical Wounds Healing by Secondary Intention Trial no. 2 (SWHSI-2)

If you wish to take part in the SWHSI-2 study, **please place your initials in each of the boxes below, sign and date this form. <u>All the information on this form will be kept confidential and will not be released to anyone outside of the research team.</u>**

<u>Participant ID:</u> ☐ ☐ ☐ ☐

Please initial each box

1.	I confirm that I have read and understand the information sheet version [*XX*] dated [*XX*] for the above study and have had the opportunity to ask any questions about the study and any questions have been answered to my satisfaction.	Initials
2.	I understand that my participation is voluntary and that I am free to withdraw at any time without giving any reason, and without my medical care or legal rights being affected.	Initials
3.	I agree to University of York's Trials Unit holding copies of my consent form, other study related documents and my contact details to allow them to send me questionnaires.	Initials
4.	I agree to my GP being informed of my participation in the study and being advised of any significant information relating to my health that comes to light during my participation.	Initials
5.	I understand that relevant sections of my hospital/GP medical notes and data collected during the study, may be looked at by individuals from University of York's Trials Unit, from regulatory authorities, the Sponsor or from the NHS Trust, where it is relevant to my taking part in the research. I give permission for these individuals to have access to my records.	Initials
6.	I consent to the storage, including electronic, of personal information for the purposes of the study by [INSERT SITE NAME] NHS Hospital, the Sponsor and University of York Trials Unit. I understand that any information that could identify me will be kept strictly confidential and that no personal information will be included in the study report or other publication.	Initials
7.	I understand that after this study has finished, the data collected from me will be securely stored in accordance with requirements of the law after which arrangements for confidential destruction will take place. Information that could identify me will be kept strictly confidential and no personal information will be included in the study report or other publication.	Initials
8.	I understand that the information collected about me will be used to support other research in the future, and may be shared anonymously with other researchers.	Initials
9.	I agree to have photographs taken of my wound during the SWHSI-2 study	Initials
10.	**I agree to take part in the SWHSI-2 Trial.**	Initials

SWHSI-2

INSERT TRUST LOGO

Print name	d d / m m / y y y y<br>_ _ / _ _ / 2 0 _ _	Signature
Name of participant (*please print*)	Date	Signature of participant

Print name	d d / m m / y y y y<br>_ _ / _ _ / 2 0 _ _	Signature
Name of person taking consent (*please print*)	Date	Signature of person taking consent

1 for participant; 1 (original) for Investigator Site File; 1 for medical notes; 1 for York Trials Unit
